# Hfq orchestrates a robust RNA-RNA interaction network in *Acinetobacter baumannii*

**DOI:** 10.1128/mbio.03231-25

**Published:** 2025-12-17

**Authors:** Valerie Intorcia, Mikaela N. Daum, Boyang Cheng, Simon L. Dove, Michael J. Gebhardt

**Affiliations:** 1 Department of Microbiology & Immunology, University of Iowa 4083, Iowa City, Iowa, USA; 2 Division of Infectious Diseases, Boston Children’s Hospital and Department of Pediatrics, Harvard Medical School 1811 , Boston, Massachusetts, USA; University of Georgia, Athens, Georgia, USA

**Keywords:** *Acinetobacter baumannii*, Hfq, post-transcriptional regulation, antibiotic resistance, small RNAs

## Abstract

Across bacteria, RNA binding proteins, such as Hfq, often play a key role in facilitating post-transcriptional regulation by chaperoning interactions between small regulatory RNAs (sRNAs) and their mRNA targets. *Acinetobacter baumannii*, a problematic gram-negative bacterial pathogen, produces over a hundred sRNAs, of which only a few have experimentally validated mRNA targets. While previous studies have identified *hfq* as a candidate essential gene in the model multidrug-resistant *A. baumannii* strain AB5075, the role of Hfq in mediating the regulatory effects of sRNAs remains largely unexplored. Here, we use RIL-seq to identify Hfq-associated sRNA-mRNA interaction partners in strain AB5075. Our results indicate that *A. baumannii* Hfq coordinates a robust network of RNA-RNA interactions. We detected RNA interactions with 98 distinct sRNA species, including nearly 40 previously undescribed sRNAs. The results further indicate that *A. baumannii* utilizes sRNAs to regulate the expression of numerous transcripts that encode virulence factors and antibiotic resistance determinants. Collectively, our study provides new insights into the regulatory potential for dozens of sRNAs in *A. baumannii* and highlights the importance of post-transcriptional regulation in this emerging pathogen.

**IMPORTANCE:**

*Acinetobacter baumannii* represents a burgeoning threat to human health and consistently ranks as a critical pathogen by the World Health Organization due to extensive antimicrobial resistance among clinical isolates. While much effort has focused on understanding how *A. baumannii* acquires antimicrobial resistance traits, our knowledge of key processes governing gene expression in this organism is lacking. In particular, very little is known regarding post-transcriptional regulation in *A. baumannii*. Here, we demonstrate that Hfq, a highly conserved RNA chaperone, coordinates the regulatory activities for nearly 100 small regulatory RNAs (sRNAs), including many that have not been described before. We also find that several Hfq-associated sRNAs directly regulate mRNA transcripts, which encode antibiotic resistance determinants and virulence factors. Collectively, our study provides evidence for the existence of a complex post-transcriptional regulatory network in *A. baumannii* and offers new insights into how the organism uses Hfq and sRNAs to coordinate gene expression.

## INTRODUCTION

The emerging bacterial pathogen, *Acinetobacter baumannii*, can cause a variety of infections in humans. As an opportunistic pathogen, *A. baumannii* frequently causes disease in the nosocomial setting and sites of infection range from surgical sites, the lung, the bloodstream, and the urinary tract (1, 2). Of major concern with these infections is the continued development of antibiotic resistance among the circulating isolates of *A. baumannii*. Indeed, numerous health organizations, including the CDC and the WHO, consistently rank multidrug-resistant (MDR) *A. baumannii* at their highest levels of concern (3, 4). Carbapenem resistance is particularly problematic in *A. baumannii*, as these antibiotics typically represent the drugs of last resort for treating infections caused by the organisms (5). Not surprisingly, contemporary MDR *A. baumannii* isolates utilize a multitude of resistance mechanisms, including both horizontally acquired resistance genes (i.e., Oxa-class carbapenemases and aminoglycoside modifying enzymes) and intrinsic resistance determinants found across the *A. baumannii* genome, including expressing porins with limited permeability for antimicrobials and multidrug efflux pumps, such as the RND-family pump encoded by the *adeIJK* genes (6).

While much effort has been devoted toward understanding the development, acquisition, and breadth of antibiotic resistance in *A. baumannii*, our understanding of how the organism coordinates gene expression remains less well understood. In particular, our understanding of post-transcriptional regulation in *A. baumannii* is lacking, despite the apparent importance of post-transcriptional regulation in the organism. Highlighting this gap in our knowledge is that while the gene encoding the key RNA binding chaperone Hfq has been identified as a candidate essential gene in the model *A. baumannii* strain AB5075 (7–9), we know very little about the scope of Hfq’s role in regulating gene expression. In the lab-adapted, antibiotic-sensitive strain *A. baumannii* ATCC 17978, the genetic interruption of *hfq* results in pleiotropic phenotypes, including growth defects, impaired carbon source utilization, increased sensitivity to environmental stressors, and decreased biofilm formation (10–12). These diverse phenotypes mirror those reported in studies examining the role of *hfq* in other bacterial species and suggest that Hfq plays a key role in maintaining the physiology of *A. baumannii*.

As an RNA chaperone, Hfq primarily functions by facilitating the interaction of small, regulatory RNA (sRNA) species with their regulatory targets, often mRNAs (13, 14). Several recent studies have sought to identify the repertoire of sRNAs produced by *A. baumannii* (15–18). These studies suggest the existence of up to several hundred sRNA species in *A. baumannii*, a number comparable to those detected in other gram-negative species. A recent publication details the results of a global RNA-RNA interactome analysis, called Hi-GRIL-seq, wherein the authors identified mRNA interaction partners for 40 sRNAs (17), hinting at the existence of a substantial post-transcriptional regulatory network in this pathogen.

A limitation to the prior RNA-RNA interactome study is that the Hi-GRIL-seq approach does not provide information on whether the detected interaction between an sRNA and its mRNA target(s) is mediated by Hfq. We report here that *hfq* is essential for the growth of several *A. baumannii* isolates and describe the repertoire of Hfq-interacting sRNAs and their regulatory RNA targets in the model strain AB5075. To this end, we employ RIL-seq (RNA interaction through ligation and sequencing), a technique developed to specifically identify RNA-RNA interaction partners mediated by an RNA-binding protein (19, 20). Using the RIL-seq approach, we have determined the Hfq-mediated RNA-RNA interactions for *A. baumannii* strain AB5075 and identified RNA interaction partners for nearly 100 sRNAs, including 38 sRNAs that have not been identified previously. Our initial follow-up analyses validate the RIL-seq findings and implicate several sRNAs that, likely in concert with Hfq, regulate the expression of highly conserved virulence factors and antibiotic resistance determinants in this emerging pathogen.

## RESULTS

### Hfq is critical for growth in multiple *A. baumannii* isolates

**Fig 1:**
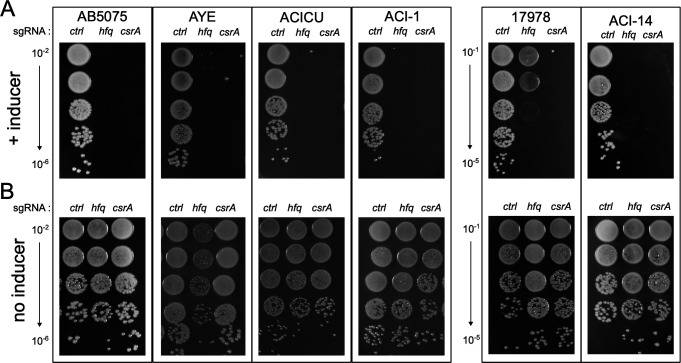
Hfq is essential in many *A. baumannii* strains. A defective *cas9* enzyme was introduced into the indicated *A. baumannii* strains at the Tn*7* attachment site. The resulting strains were transformed with plasmids driving constitutive expression of the indicated guide RNAs (sgRNA): *ctrl*, control sgRNA targeting *mCherry*; *hfq*, sgRNA targeting the *hfq* open reading frame; *csrA*, sgRNA targeting the *csrA* open reading frame. Colonies were serially diluted in sterile PBS, and the indicated dilutions were plated on (**A**) LB plates with 100 ng/mL of anhydrotetracycline or (**B**) LB plates. Plates were incubated overnight at 37°C (≈ 16 h) and photographed.

Hfq is a candidate essential protein in *A. baumannii* strain AB5075-UW (7, 8), and we previously demonstrated that depletion of Hfq via CRISPR interference (CRISPRi) leads to impaired growth in this strain background (9). Interestingly, there are several publications that have characterized *hfq* mutants in the commonly studied and antibiotic-sensitive *A. baumannii* strain ATCC 17978 (10, 12, 21). We thus aimed to determine if the essentiality of *hfq* in the AB5075-UW background is a strain-specific phenomenon and whether *hfq* is also essential in other *A. baumannii* isolates. To answer this question, we introduced a defective Cas9 (dCas9) allele in single copy at the Tn*7* attachment site in several *A. baumannii* isolates, including commonly studied strains (ATCC 17978, AYE, and ACICU) and two clinical isolates (ACI-1, ACI-14). Once constructed, we transformed the dCas9 strains with a plasmid driving the constitutive expression of a single guide RNA (sgRNA) targeting the non-template strand of the *hfq* coding sequence, or a control sgRNA targeting the coding sequence of *mCherry*, which is absent from the *A. baumannii* genome (7, 9). As shown in Fig. 1, CRISPRi-mediated depletion of *hfq* severely impaired growth on solid media for all the strains tested, except ATCC 17978 (Fig. 1). It is notable, however, that the growth of strain 17978 expressing the *hfq* sgRNA was reduced relative to cells harboring the control sgRNA plasmid. As an additional control, we also targeted *csrA*, which is essential for *A. baumannii* to grow on rich media (22, 23). These data indicate that *hfq* is likely essential in multiple *A. baumannii* isolates and further highlight the importance of post-transcriptional regulation in the species, as *hfq* and *csrA* are both critical for growth.

### RIL-seq analysis of Hfq-associated RNAs in *A. baumannii*

**Fig 2:**
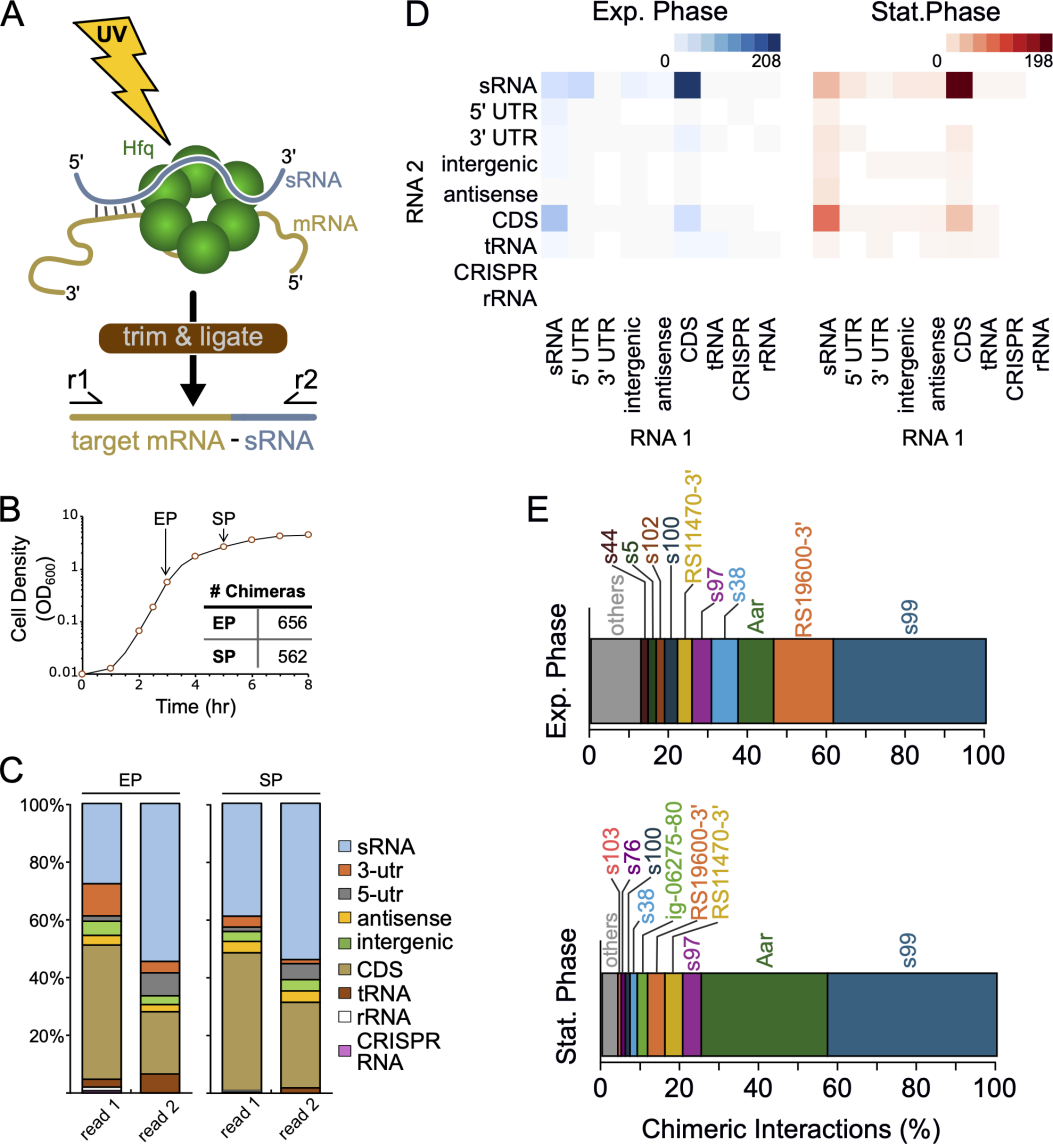
RIL-seq with Hfq identifies RNA-RNA interactions in *A. baumannii*. (**A**) Schematic of the RIL-seq technique. Triplicate cultures of wild-type *A. baumannii* AB5075 and its *hfq-V* derivative were grown to exponential phase (OD600 ≈ 0.6) and early stationary phase (OD600 ≈ 2.0). Cells were exposed to ultraviolet radiation to form cross-links between proteins and nucleic acids prior to immunoprecipitation of Hfq-RNA complexes. These complexes were then treated with RNases and RNA ligase to join neighboring RNA ends. Following protease treatment and purification of RNA, the chimeric RNAs were identified by paired-end Illumina sequencing. (**B**) Representative growth curve of AB5075 *hfq-V* used for RIL-seq the experiment. The inset depicts the number of chimeras detected in each growth phase. (**C**) Distribution of types of RNA detected in the RNA1 (read 1) and RNA2 (read 2) positions of RIL-seq chimeras for each data set. EP, exponential phase; SP, stationary phase. (**D**) Quantification of types of chimeric species detected among the RNA-RNA interactions in the exponential (Exp.) or stationary (Stat.) phases RIL-seq data sets. Heatmaps depict the frequency for each type of interaction detected. Columns represent the annotation type for the RNA1 position of the chimera, and rows represent the annotation type for the RNA2 position. (**E**) Relative abundance of the sRNAs detected in chimeras in RIL-seq data sets in the exponential (Exp.) or stationary (Stat.) phase of growth. Data represent the relative abundance for the top 10 most abundant sRNA detected in each growth phase. Abundance determined by dividing the number of chimeric fragments involving a particular sRNA by the total number of chimeric fragments containing any sRNAs, independent of its read position (i.e., RNA1 or RNA2). Note that chimeric fragments consisting of two sRNAs were counted twice, and the total number of chimeras was updated accordingly.

Given that Hfq mediates post-transcriptional regulation through its ability to chaperone interactions between two RNA species, we sought to determine the repertoire of interacting RNA transcripts mediated by Hfq in *A. baumannii* strain AB5075. We first constructed an AB5075 derivative, wherein the chromosomal allele of *hfq* was modified to specify a vesicular stomatitis virus G-protein (VSV-G) epitope at its C-terminus (Hfq-V). Analysis of the growth kinetics for the AB5075 Hfq-V strain showed a slight extension of the lag period in the Hfq-V strain, after which the growth rate was similar to wild-type AB5075 (Fig. S1). We next applied the RIL-seq experimental approach on triplicate cultures of Hfq-V cells grown in rich medium (LB) at 37°C to mid-to-late exponential phase (EP, OD_600_ ≈ 0.6) and early stationary phase (SP, OD_600_ ≈ 2.0). Wild-type AB5075 cells, which do not express any VSV-G tagged proteins, served as a negative control for the experiment. As described in the Methods section and elsewhere (19, 24), cells collected at the indicated growth stages were washed and resuspended in PBS before subsequent exposure to UV irradiation to cross-link nucleic acids and proteins. The cross-linked cells were lysed, and Hfq-V-RNA-RNA complexes were immunoprecipitated with antibodies targeting the VSV-G epitope. The resulting Hfq-V-RNA-RNA complexes were subjected to limited RNase digestion, followed by RNA ligation to form covalent bonds between interacting RNAs on Hfq, and treated with protease prior to RNA isolation (see schematic in Fig. 2). The purified RNA, containing a mixture of both single and chimeric RNA species, was then subjected to Illumina sequencing library construction and paired-end sequencing. Through mapping and bioinformatic analyses, the ends of each RNA fragment were independently mapped to identify fragments originating from two distinct locations in the genome. Previous RIL-seq experiments conducted for Hfq from other bacterial species have consistently demonstrated that reads mapped as the second read on the sequencer (i.e., RNA2) correspond to RNAs interacting with the proximal face of Hfq (typically sRNAs), while reads in the RNA1 position indicate interaction with the distal face of Hfq (often mRNAs) (20, 25–27). Consistent with these previous findings, in both the exponential and stationary phase RIL-seq libraries, at least 50% of the reads mapping as RNA2 appear to originate from the 3′-untranslated regions (UTR) of annotated genes or to sRNA annotations (Fig. 2C). Similarly, over half of the RNA1 reads mapped to mRNA or the 5′-UTR of annotated genes (Fig. 2C). Correlation analyses indicated that the intra-library variance was lower than expected by chance, and we thus combined the triplicate libraries into a single data set and identified those chimeric fragments that were statistically significant (Fig. S2) (24). Additionally, we observed no significant difference between the three replicates for chimeric reads mapping to sRNA annotations based on read position (i.e., reads mapping to sRNAs in the read 1 or read 2 position) for either growth phase (Fig. S2), suggesting that the distribution of types of RNA transcripts bound by Hfq was similar across the various biological samples and sequencing libraries. As noted above, cells of wild-type AB5075 were subjected to the same RIL-seq experimental, sequencing, and bioinformatic analyses as a control experiment. Consistent with previous studies, a small number of chimeric RNA fragments were identified in these control data sets. To account for this, we applied an additional threshold to only include those chimeras detected more frequently than 90% of the chimeras detected in the control data sets. Our final Hfq-V data sets thus include those chimeras detected more than 13 and 11 times, respectively, for the exponential and stationary phase data sets.

In total, RIL-seq for Hfq-V in *A. baumannii* AB5075 identified 656 chimeras in exponential phase and 562 in the stationary phase (Data set S1). Many of the RNA-RNA chimeras appear to originate from mRNA-sRNA interactions, with mRNAs comprising the 5′ portion and sRNAs the 3′ portion of the chimeras (Fig. 2C and D). Between the two RIL-seq data sets, we detected 98 distinct sRNAs (73 in exponential phase, 80 in stationary phase), which comprise both previously known sRNAs and novel sRNAs that have not been identified previously (Data set S1, Table S1). While most of the sRNAs were detected as the second read in the chimeras (i.e., RNA2), numerous chimeras were identified with an sRNA in the read 1 position (Fig. 2C and D). The independently transcribed sRNA, sRNA99 (also called ABUWs076 by ref. 15), was the most prevalent sRNA detected in chimeras for both the exponential and stationary phase data sets (Fig. 2E). Interestingly, sRNA99 was most frequently detected as the first RNA in the chimera (i.e., the RNA1 location) with 85 and 77 chimeras in exponential phase and stationary phase, respectively, and was considerably less frequently found as the second RNA in chimeras, being identified seven times in the exponential phase data set and 12 times in stationary phase (Fig. S3). The behavior of sRNA99 in our RIL-seq experiment is reminiscent of the *Pseudomonas aeruginosa* sRNA CrcZ, which was also predominantly found as the RNA1 in Hfq chimeras by RIL-seq (19). Like CrcZ, the primary sequence of sRNA99 includes three potential A-rich motifs (AAN motifs) that specifically associate with the distal surface of Hfq (28, 29), which may explain why sRNA99 was primarily identified as the first read in chimeric interaction pairs (Fig. S3). In the *P. aeruginosa* RIL-seq data set, CrcZ was the most highly abundant sRNA detected on Hfq when the authors analyzed their data for single fragments. However, when we analyzed the *A. baumannii* RIL-seq data for single fragments, the most abundant RNA that copurified with Hfq was not sRNA99, but rather was OmpZ, a previously undescribed sRNA that appears to be derived from the 3′-untranslated region (UTR) of a transcript encoding an OmpW-family outer membrane protein (locus tag: *ABUW_RS17440*; old locus tag: *ABUW_3583*). OmpZ was detected in 23 and 12 chimeras in the exponential phase and stationary phase data sets, respectively. Remarkably, OmpZ accounted for over 60% and 40% of the Hfq-associated single RNA fragments detected in exponential phase and stationary phase data sets, respectively (Fig. S4). The high relative abundance of OmpZ on Hfq may suggest that OmpZ has high affinity for Hfq.

Of the coding transcripts (i.e., those mapping to mRNAs and 5′-UTRs) identified in the RNA1 position, the most prevalent predicted functions included metabolism and transporters (Table 1). These top categories were shared in both the exponential and stationary phase data sets, with transcripts encoding functions in transcription regulation and translation also being prevalent. In the exponential phase data set, we detected several transcripts related to the type IV pilus apparatus, which is consistent with these genes being more highly expressed during this growth phase (30). In the stationary phase data set, we also detected many RNA transcripts encoding stress response functions and antibiotic resistance genes (Table 1). Further, consistent with the essential nature of *hfq* in *A. baumannii*, we detected numerous transcripts encoding essential genes (52 and 23 in exponential and stationary phase data sets, respectively). We also cross-referenced the mRNAs identified by Hfq RIL-seq with those of recent transposon-insertion sequencing experiments that sought to identify genetic requirements for antibiotic resistance (31) and/or virulence (7, 8, 32) in *A. baumannii*. These analyses revealed that Hfq interacts with numerous RNA transcripts required for virulence and antibiotic resistance in *A. baumannii* (Table S1).

**TABLE 1:** Functional characterization of RIL-seq data^*a*^

Functional category	Number of targets
Exponential phase	
Metabolism	65
Hypothetical proteins	64
Transport	49
Translation	21
Transcription regulation	18
Peptidoglycan/cell wall	17
Type IV pilus	13
DNA replication/repair	18
Protein folding	8
Metal ions	7
Stationary phase	
Hypothetical proteins	55
Metabolism	51
Transport	27
Transcription regulation	14
Stress response	13
Translation	8
DNA replication/repair	7
Peptidoglycan/cell wall	6
Signal transduction	5
Cell division	4

^*a*^ Analysis based on chimeras in which the RNA1 position mapped to either a 5′-UTR or open reading frame.

### Several *A. baumannii* sRNAs require Hfq for their stability

**Fig 3:**
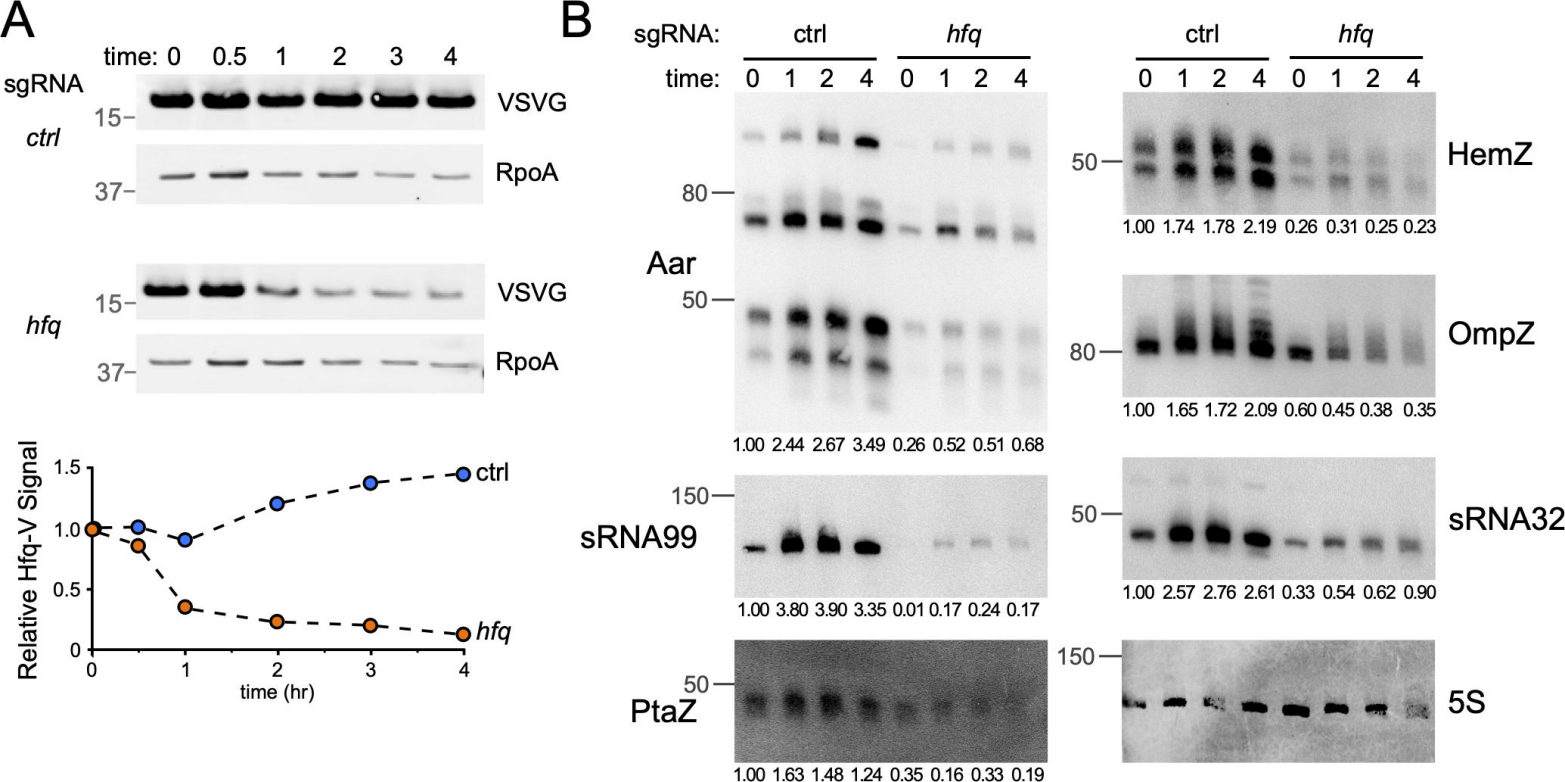
Several sRNAs detected by RIL-seq require Hfq for stability. CRISPR interference targeting of *hfq* leads to reduction in (**A**) Hfq protein abundance and (**B**) RNA abundance for several sRNAs identified by RIL-seq. AB5075 *hfq-VSV-G att*Tn*7-dcas9* (**A**) or AB5075 *att*Tn*7-dcas9* (**B**) cells harboring plasmids expressing control (*ctrl*; targeting the open reading frame for *mCherry*) or *hfq* targeting sgRNAs were induced for *dcas9* expression at early exponential phase of growth (OD_600_ ≈ 0.4). At the indicated times (in hours), cells were collected for Western blot analysis (panel A) or northern blot analysis (panel B). In panel **A**, whole-cell lysates were separated by SDS-PAGE, transferred to a PVDF membrane, and probed with anti-VSV-G antibodies to detect Hfq-V protein abundance and anti-RNA-polymerase subunit alpha (RpoA) as a loading control (top panels). The graph depicts the relative abundance of Hfq-V in the depletion and control strains as a function of time (lower panel). In panel **B**, total RNA collected at the indicated time points was separated on a denaturing acrylamide gel, transferred to a positively charged nylon membrane, and probed with P-32 end labeled oligonucleotides for the indicated sRNAs. An IR-DYE-700 labeled oligonucleotide probe complementary to the sequence of the 5S rRNA gene was used as a loading control. In panel **B**, the same membrane was stripped and re-probed as necessary. Numbers below blot panels indicate the relative abundance of the sRNA (compared to the 5S rRNA signal) for each time point compared to that of the control sgRNA signal at time 0.

In multiple species of gram-negative bacteria, sRNAs often require Hfq for their stability (19, 25, 33). As *hfq* is essential in *A. baumannii* strain AB5075-UW (Fig. 1; references 7, 8), we have been unable to delete the chromosomal copy of *hfq* to assess its role in maintaining the stability of sRNAs identified by our RIL-seq study. As an alternative to creating a deletion, we reasoned that reducing the abundance of the Hfq protein using CRISPRi would allow us to determine if Hfq is required for the abundance or stability of sRNAs. In the Hfq-V strain background used for RIL-seq, we assessed the efficiency of CRISPRi-mediated depletion of Hfq by Western blot in cells harboring dCas9 at the Tn*7* attachment site along with either the *hfq* sgRNA plasmid or mCherry sgRNA plasmid as a control. Induction of dCas9 in cells harboring the *hfq-*targeting sgRNA resulted in the reduction of Hfq levels to approximately 10% of that in the uninduced condition or in cells expressing the control sgRNA (Fig. 3). We next collected total RNA from the AB5075-*dCas9* strain following induction in the presence of either the control sgRNA or the *hfq*-targeting sgRNA and performed northern blot analyses to assess sRNA stability in cells depleted of Hfq. We conducted northern blots for several sRNAs identified as Hfq-associated in our RIL-seq experiment. As shown in Fig. 3B, we found that several sRNAs are less abundant when Hfq levels are reduced, including the three sRNAs described in further detail below: Aar, PtaZ, and HemZ. Collectively, these results indicate that in *A. baumannii*, Hfq is required for the stability and/or expression of several of the sRNAs with which it interacts, consistent with findings from other bacterial species, including *E. coli* (25, 33) and *P. aeruginosa* (19).

### Aar regulates expression of RsuA and CarO

**Fig 4:**
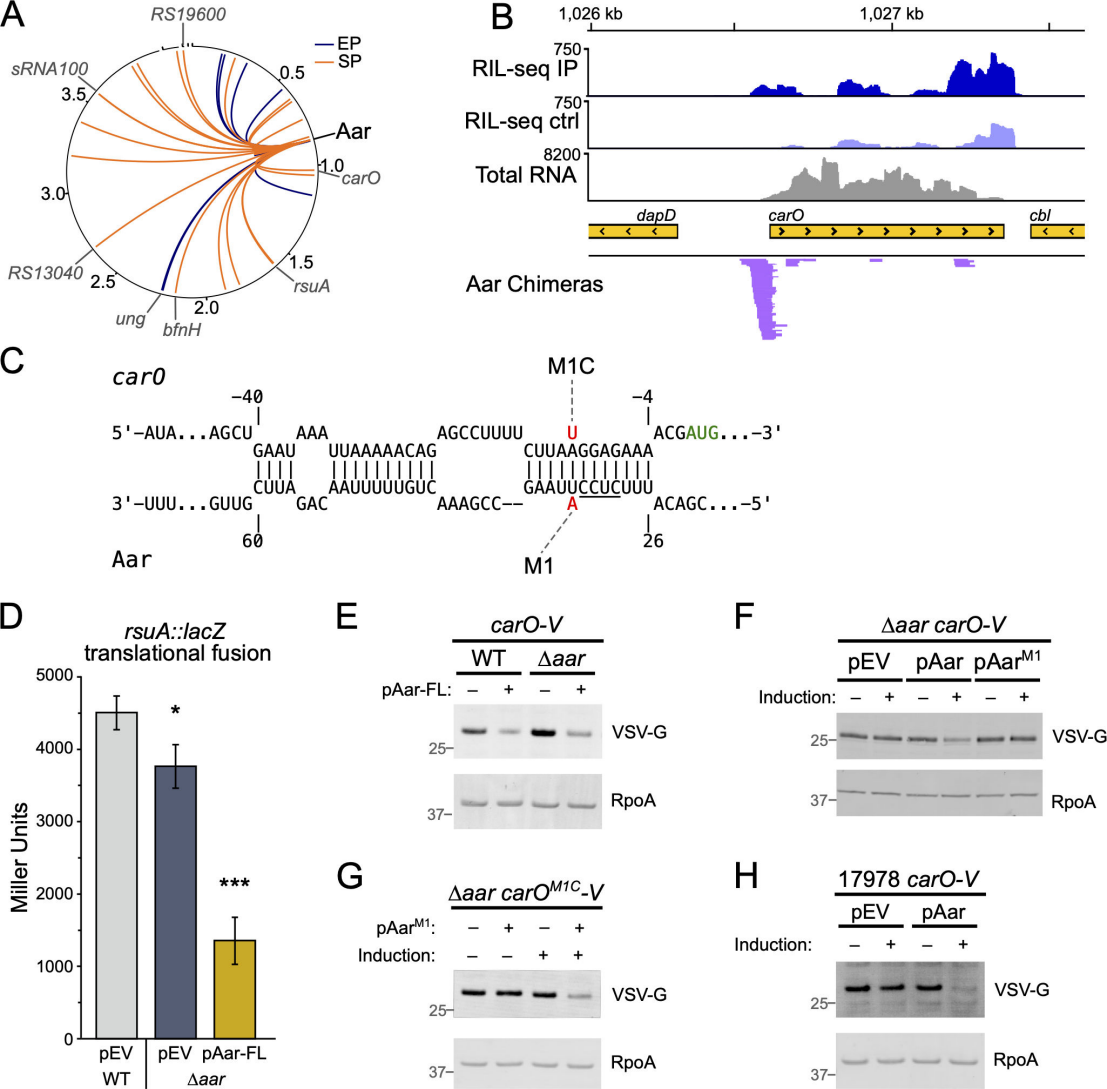
Aar regulates expression of CarO and RsuA. (**A**) Circos (37) plot of RIL-seq chimeras containing Aar. Circle represents the chromosome and three plasmids of AB5075-UW, and numbers indicate genome position (in Megabases). Aar location is indicated in black text, and gray text indicates RNA targets also identified by Hi-GRIL-seq (17). Links between Aar and other positions indicate chimeras detected in exponential phase (EP, blue) and stationary phase (SP, orange). (**B**) RIL-seq data for *carO* region. RIL-seq IP panel indicates read depth detected in a representative stationary phase IP sample. RIL-seq ctrl panel indicates read depth detected in a representative stationary phase control sample. Total RNA panel indicates read depth in a representative total RNAseq sample. Aar chimeras panel indicates position of chimeras detected containing Aar as RNA2. Yellow bars indicate annotations; the internal black arrows indicate the direction of transcription. For IP, control, and Total RNA tracks, only reads mapping to the forward strand are shown. (**C**) IntaRNA (38) prediction of the interaction between *carO* and Aar. The *carO* start codon is indicated in green text. Locations of the Aar-M1 and *carO*-M1C mutant alleles are indicated. Numbers indicate the nucleotide position relative to the start codon of *carO* (above) and first nucleotide of Aar (below). The Aar “seed” sequence (CUCC) previously identified by Hamrock and colleagues (17) is underlined. (**D**) β-galactosidase activity (in Miller Units) of AB5075 WT cells or its ∆*aar* derivative containing a translational *rsuA::lacZ* reporter integrated at the Tn*7* attachment site in the presence of an empty vector (pEV) or Aar expression vector (pAar-FL). The β-galactosidase assay was repeated independently at least twice with biological triplicate cultures. Data from a single representative experiment are plotted as the mean activity with error bars representing one SD of the mean. Significance was assessed by a two-tailed *t*-test comparing the activity to that of WT cells with empty vector. Asterisks indicate significant differences with *P*-value ≤ 0.05 (*), *P*-value ≤ 0.001 (***). (**E–G**) Western blot analyses to detect CarO-V in cells grown to early stationary phase (6 h, OD_600_ ≈ 2.0) in the presence or absence of the Aar plasmid and/or inducer. In panels **E and F**, cells encode an allele of *carO* with a C-terminal VSV-G epitope sequence at the chromosomal *carO* locus in wild-type AB5075 (WT) or an *aar* deletion mutant background (∆*aar*). In panel **G**, cells encode the *carO^M1C^-V* allele in the ∆*aar* background. In panel **H**, cells encode an allele of *carO* with a C-terminal VSV-G epitope sequence in otherwise wild-type *A. baumannii* ATCC 17978. For Western blotting, whole-cell lysates prepared from cells of the indicated strains/plasmids were resolved by SDS-PAGE, transferred to PVDF membranes, and analyzed by Western blot with antibodies recognizing the VSV-G epitope (VSV-G) or the RNA polymerase Alpha subunit (RpoA). Markers in gray text indicate the position of protein size standards (in kDa). The predicted molecular weight of CarO-V is 26 kDa, and RpoA is 38 kDa. Western blot experiments were completed with biological triplicate cultures and repeated independently at least twice. Data from a single experiment are shown. In panels **E and G**, samples in lanes with a dash (–) were collected from cells harboring an empty vector (pMJG598). Where indicated in panels **F and H**, cultures included 50 ng/mL anhydrotetracycline (aTc) as an inducer. pEV, empty vector pMJG598, pAar-FL, pMJG598 harboring Aar under control of its native promoter, pAar, pMJG598 with Aar under control of the P*_tetA_* promoter, pAar-M1, pMJG598 with Aar-M1 allele under control of the P*_tetA_* promoter.

As noted above, a key challenge posed by *A. baumannii* is the ongoing development of extensive antibiotic resistance, with carbapenem resistance being particularly problematic (5). A highly abundant sRNA in our RIL-seq data set is Aar (Fig. 2E), an independently transcribed and highly conserved sRNA in *A. baumannii* (15). Aar was initially characterized in *Acinetobacter baylyi*, where it was shown to play a role in amino acid regulation (34). More recently, work from the Kröger laboratory identified Aar as a prominent *A. baumannii* sRNA in a global RNA-RNA interactome study, where the authors identified interacting RNA transcripts independently of any RNA-binding proteins (17). In agreement with these previous reports, we found that Aar exhibits higher expression as cells enter stationary phase (Fig. 3) and found that Aar interacts with 29 distinct RNA transcripts across the two RIL-seq data sets: 12 in exponential phase and 21 in stationary phase, with 4 common to both growth phases (Fig. 4). Seven of the Aar-target interactions were also found in the previous study from Kröger and colleagues (17). Among the targets common to both data sets are *rsuA*, which encodes a predicted RNA pseudouridine synthase, and *carO*, which encodes an outer membrane porin that has long been associated with carbapenem resistance in *A. baumannii* clinical isolates (35, 36).

To test whether Aar can exert regulatory effects on any of these targets, we first created an *aar* deletion strain, wherein the Aar nucleotide sequence was replaced with that of the tR′ transcription terminator sequence, hereafter referred to as *∆aar*. By northern blot analysis, we did not detect any Aar signals in RNAs collected from the *∆aar* strain (Fig. S5). Also of note are the multiple Aar-probe-reactive RNA species visible by northern blot analysis (Fig. 3B; Fig. S5). These RNA species are specific to Aar, as they are absent from the *∆aar* strain and have been observed previously by Hamrock and colleagues (17). It is unclear whether the multiple Aar-specific RNA transcripts arise from multiple promoters in the Aar region, RNA processing events of the mature Aar sRNA, or degradation intermediates of the longer Aar transcript. In addition to the ∆*aar* deletion strain, we created two Aar expression plasmids, including a version that included the native Aar promoter (pAar-FL) and a second plasmid with the Aar transcription start site encoded at the corresponding +1 site of a tetracycline-inducible promoter (pAar). Both Aar expression plasmids give rise to the same Aar-probe-specific RNA species when analyzed by northern blot (Fig. S5). We next created a translational *lacZ* fusion reporter for *rsuA*. Introduction of a plasmid carrying the Aar sequence along with its own promoter (pAar-FL) resulted in decreased activity of the *rsuA::lacZ* translational fusion, suggesting that Aar regulates expression of this RNA modification enzyme (Fig. 4D). Importantly, the Aar expression plasmid did not appreciably impact the activity of a transcriptional *rsuA::lacZ* reporter (Fig. S5). We also performed an *in silico* RNA-RNA interaction prediction using IntaRNA (38) to identify a potential RNA-RNA interaction site between Aar and the *rsuA* mRNA, revealing an extensive potential interaction region between the two RNAs (Fig. S5). The predicted interaction site likely overlaps with the *rsuA* ribosome binding site, and the portion of Aar predicted to interact with RsuA encompasses the same seed sequence required for Aar-mediated regulation of both the *carO* and *bfnH* mRNAs (Fig. 4C) (17). Taken together, these observations suggest that Aar exerts post-transcriptional control over RsuA expression.

The RIL-seq data indicated that Aar interacts with the *carO* transcript near the translation initiation region (Fig. 4B). To test whether the putative interaction between Aar and the *carO* mRNA we had identified was regulatory in nature, we constructed strains of both WT AB5075 and an isogenic ∆*aar* derivative, each expressing a *carO* allele with a C-terminal VSV-G epitope from the native *carO* locus (CarO-V). We observed a modest increase in CarO-V abundance in cells lacking Aar (∆*aar*), and when Aar was provided from a multi-copy plasmid (pAar), we observed a significant reduction in CarO-V abundance, suggesting that Aar regulates CarO expression (Fig. 4E). As noted above, Kröger and colleagues detected Aar-*carO* mRNA interactions and showed that mutation of a four-nucleotide seed sequence in Aar (CUCC, spanning Aar nucleotides 29–32 and underlined in Fig. 4C) impaired the ability of Aar to regulate *carO* translation (17). To confirm that the interaction between Aar and the *carO* mRNA is direct, we performed further mutational analyses of the nucleotides in the predicted Aar-CarO interaction and created an Aar allele harboring a single nucleotide change, U33A, which we refer to here as Aar-M1 (Fig. 4C). The M1 mutation is immediately upstream of the previously identified Aar seed sequence, and the Aar-M1 mutation abrogated the repression of CarO (Fig. 4F). We then introduced a compensatory mutation in the *carO* 5′-untranslated region (*carO*^M1C^), which restored the ability of the Aar-M1 mutant to repress the translation of CarO (Fig. 4G). These results thus confirm that Aar controls *carO* expression through a direct interaction between Aar and the *carO* mRNA transcript. We next asked whether the regulatory link between Aar and CarO was conserved in other *A. baumannii* isolates. We created a derivative of *A. baumannii* strain ATCC 17978 specifying the same VSV-G epitope tag at the C-terminus of the *carO* gene. Much like our observations in the AB5075 background, ectopic expression of Aar in the 17978 strain background led to a similar decrease in CarO abundance, as assessed by Western blotting (Fig. 4H). This observation suggests that Aar likely regulates, through a direct interaction, the expression of CarO across the *A. baumannii* species.

Given the association between CarO and carbapenem resistance in clinical isolates, we next assessed the phenotypic consequence of Aar-mediated repression of CarO by assessing the carbapenem resistance profile for strains with altered Aar levels (i.e., ∆*aar* and cells harboring pAar). In the AB5075 strain background, no overt differences in the resistance profile for either imipenem or meropenem were observed, even in a strain background harboring a deletion of *oxa23*, which encodes an Oxa23-family carbapenemase known to be a main determinant of carbapenem resistance in strain AB5075 (Table 2) (39). Surprisingly, we also did not observe differences in carbapenem susceptibility in a *∆carO* deletion strain in the ATCC 17978 background, nor were there differences in susceptibility to antimicrobials not associated with CarO, including ceftazidime (a cephalosporin) and gentamicin (an aminoglycoside). In the AB5075 background, deletion of *carO* caused a slight decrease in resistance to tetracycline, although the relevance of this modest change in zone of inhibition is unclear (Table 2). These results indicate that CarO does not play a role in carbapenem resistance in either AB5075 or ATCC 17978. While our study was in progress, McCalla and colleagues reported a similar observation in a different set *A. baumannii* strain backgrounds, further supporting that CarO may not have a prominent role in carbapenem resistance (40).

**TABLE 2:** Disc diffusion assay results for strains lacking *carO^a^*

Strain	Zone of inhibition (mm)				
	Imipenem	Meropenem	Ceftazidime	Gentamicin	Tetracycline
AB5075	9	8	7	11	16.3
AB5075 ∆*carO*	10	8	7	11	18
AB5075 ∆*oxa23*	20	14	7	11	16
AB5075 ∆*oxa23* ∆*carO*	20	14	7	11	17
ATCC 17978	21	21	16	24	18
ATCC 17978 ∆*carO*	21	21	16	24	16

^*a*^ Zone of inhibition reported as the average (in mm) of three biological triplicate samples. Imipenem and meropenem discs contained 10 ng of antibiotic; ceftazidime disc contained 30 ng of antibiotic; gentamicin and tetracycline discs were impregnated with 250 and 25 ng of each antibiotic, respectively.

### The novel sRNA PtaZ regulates BasJ expression

**Fig 5:**
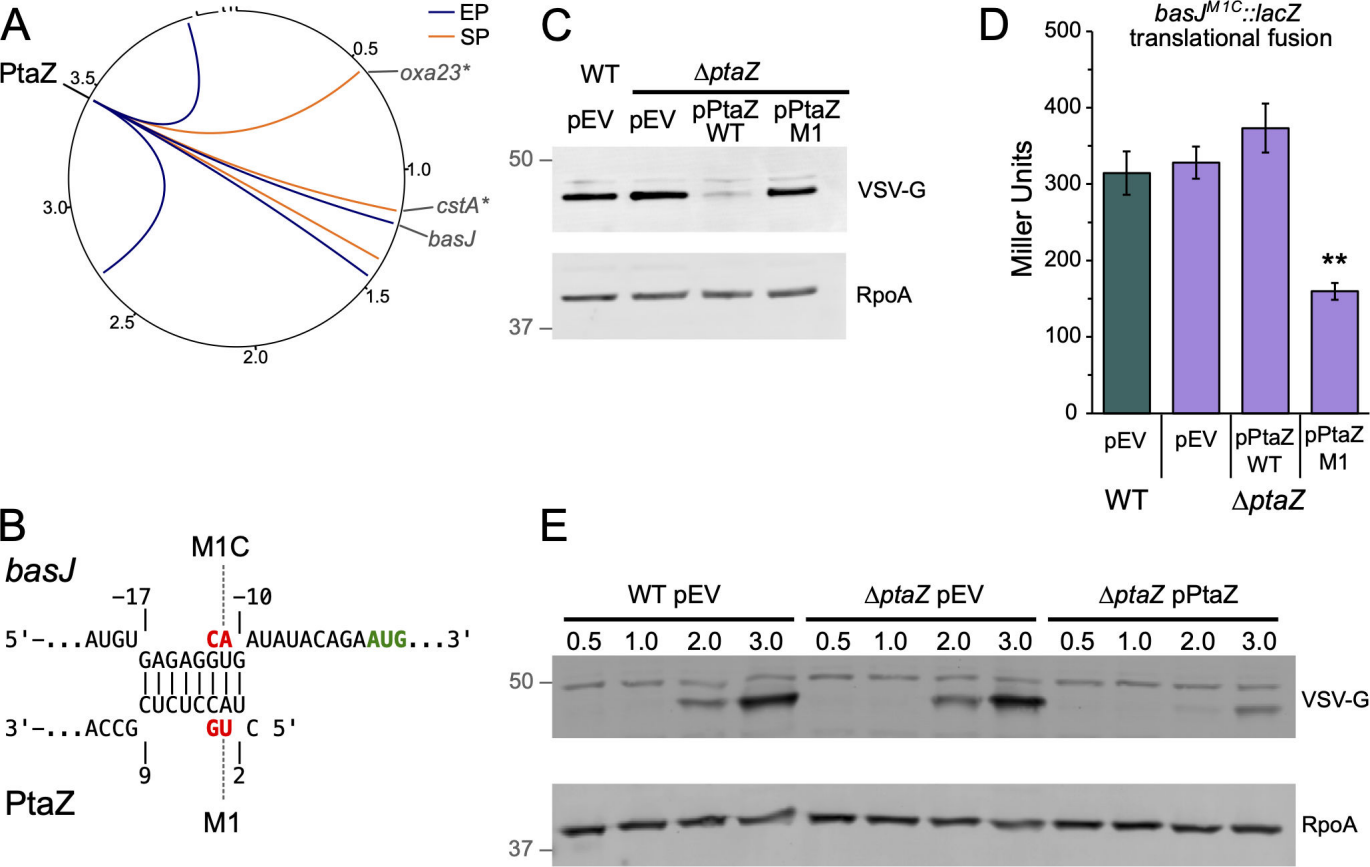
sRNA PtaZ directly regulates expression of BasJ. (**A**) Circos (37) plot of RIL-seq chimeras containing PtaZ. Circle represents the chromosome and three plasmids of AB5075-UW, numbers indicate genome position (in megabases). PtaZ location is indicated in black text, gray text indicates select RNA targets, and asterisks indicate targets detected in both exponential phase (EP) and stationary phase (SP) data sets. Links between PtaZ and other positions indicate chimeras detected in EP (blue) and SP (orange). (**B**) IntaRNA (38) prediction of the interaction between *basJ* and PtaZ. The *basJ* start codon is indicated in green text. Locations of the PtaZ-M1 and *basJ*-M1C mutant alleles are indicated. Numbers indicate the nucleotide position relative to the start codon of *basJ* (above) and first nucleotide of PtaZ (below). (**C**) Western blot analysis to detect BasJ-V. WT or ∆*ptaZ* mutant cells harboring the *basJ-V* allele and the indicated plasmid were grown in M9-succinate medium for 6 h (OD_600_ ≈ 0.3) in the presence of inducer (5 ng/mL aTc). The membranes were probed with antibodies recognizing VSV-G or RpoA. Markers in gray text indicate the position of protein size standards (in kDa). The predicted molecular weight of BasJ-V is 45 kDa and RpoA is 38 kDa. (**D**) β-galactosidase activity (in Miller Units) of AB5075 WT cells or its ∆*ptaZ* derivative containing a translational *basJ^M1C^::lacZ* reporter integrated at the Tn*7* attachment site in the presence of an empty vector (pEV), wild-type PtaZ expression vector (pPtaZ), or PtaZ-M1 mutant allele expression vector (pPtaZ-M1). The β-galactosidase assay was repeated independently at least twice with biological triplicate cultures. Data from a single representative experiment are plotted as the mean activity with error bars representing one SD of the mean. Significance was assessed by a two-tailed *t*-test comparing the activity to that of WT cells with empty vector. Asterisks indicate significant differences with *P*-value ≤ 0.01 (**). (**E**) Western blot analysis following switch from iron-replete (LB) to iron-limited (M9-succinate) media. Cultures of WT BasJ-V or ∆*ptaZ* BasJ-V containing the indicated plasmids were grown for 3 h in LB, washed in M9-salts, and resuspended in M9-succinate medium containing 5 ng/mL aTc and grown with shaking at 37°C. At the indicated time points (in hours), samples were collected and analyzed by Western blot. Western blot experiments were repeated independently at least three times. Data from a single experiment are shown.

A key aspect of *A. baumannii* infection biology is iron acquisition (41). Like many other bacterial pathogens, *A. baumannii* isolates produce multiple iron-scavenging siderophores (42). Chief among the numerous siderophores produced by *A. baumannii* is acinetobactin, which was recently shown to be critical for scavenging iron from the mammalian host in *A. baumannii* strain 17978 (43). In our stationary-phase RIL-seq data set, we detected chimeras between a novel small RNA, which we named PtaZ and the transcript for *basJ*, which encodes an isochorismate synthetase enzyme involved in generating a key building block of acinetobactin (44). In our RIL-seq experiments, PtaZ interacted with nine different RNA transcripts, including three in the exponential phase data set and six in the stationary phase; two of the PtaZ-target chimeras were present in both data sets (Fig. 5). Based on the RIL-seq data, PtaZ appears to be derived from the 3′ end of the longer *ackA-pta* mRNA transcript, whose gene products—acetate kinase (*ackA*) and phosphate acetyltransferase (*pta*)—are involved in overflow metabolism in other bacteria (45, 46).

To investigate the synthesis of PtaZ, we performed a 5′-RACE (rapid amplification of cDNA ends) experiment. The results indicate that PtaZ begins 20 nucleotides downstream of the stop codon for the *pta* gene product (Fig. S6), suggesting that PtaZ is approximately 41 nucleotides in length, which is consistent with our northern blotting results (Fig. 3; Fig. S6). PtaZ-*basJ* chimeras were detected in the stationary phase data set, and IntaRNA predictions indicated that PtaZ may interact directly with the *basJ* mRNA at or near the ribosome binding site for *basJ* (Fig. 5B). Consistent with this prediction, ectopic expression of PtaZ reduced the abundance of a VSV-G epitope-tagged copy of BasJ at the native locus (BasJ-V) as measured by Western blot (Fig. 5C). When we mutated two PtaZ nucleotides in the predicted PtaZ-*basJ* interaction region (referred to as PtaZ-M1), we no longer observed translational repression of BasJ. Our initial attempts to generate a compensatory mutation in the *basJ* target to restore the interaction with PtaZ-M1 were unsuccessful, as we were unable to detect the M1C-BasJ-V by Western blot. We suspect that the compensatory mutation we introduced overlaps with the ribosome binding site on the *basJ* transcript, impairing translation. This possibility is supported by the results of β-galactosidase assays with *basJ::lacZ* translational fusion reporters, where the compensatory *basJ-M1C* mutation results in approximately a 10-fold reduction in *basJ* reporter activity (Fig. S6). Despite this reduction in activity, we were still able to demonstrate that the PtaZ-M1 mutant, which does not regulate wild-type *basJ*, represses the translation of a BasJ-M1C reporter harboring the compensatory mutations (Fig. 5D), confirming a direct interaction between PtaZ and BasJ. As an additional control, we created a *basJ::lacZ* transcriptional reporter. In contrast to the *basJ::lacZ* translational reporter, ectopic expression of PtaZ actually led to an increase in reporter activity (Fig. S6). Thus, because ectopic PtaZ appears to increase the activity of the *basJ* promoter, the negative regulatory effect on expression of the translational reporter that we report may be an underestimate.

We next investigated whether PtaZ impacts the ability of *A. baumannii* to adapt to changes in iron availability by monitoring the accumulation of BasJ-V following a shift from iron-replete (LB media) to iron-poor conditions (M9-succinate media). As expected, accumulation of BasJ-V in M9-succinate was dependent on the concentration of iron in the media, as the addition of 30 µM FeCl_3_ abrogated BasJ-V accumulation (Fig. S6). Following the shift to iron-poor conditions, BasJ-V accumulation was delayed in cells ectopically expressing a plasmid-borne copy of *ptaZ* compared to wild-type cells (Fig. 5E). Taken together, these data indicate that PtaZ directly represses the translation of BasJ and, in so doing, may regulate the synthesis of a virulence-required siderophore in *A. baumannii*.

### The expression of AdeI is regulated by HemZ, a novel 3′-UTR derived sRNA

**Fig 6:**
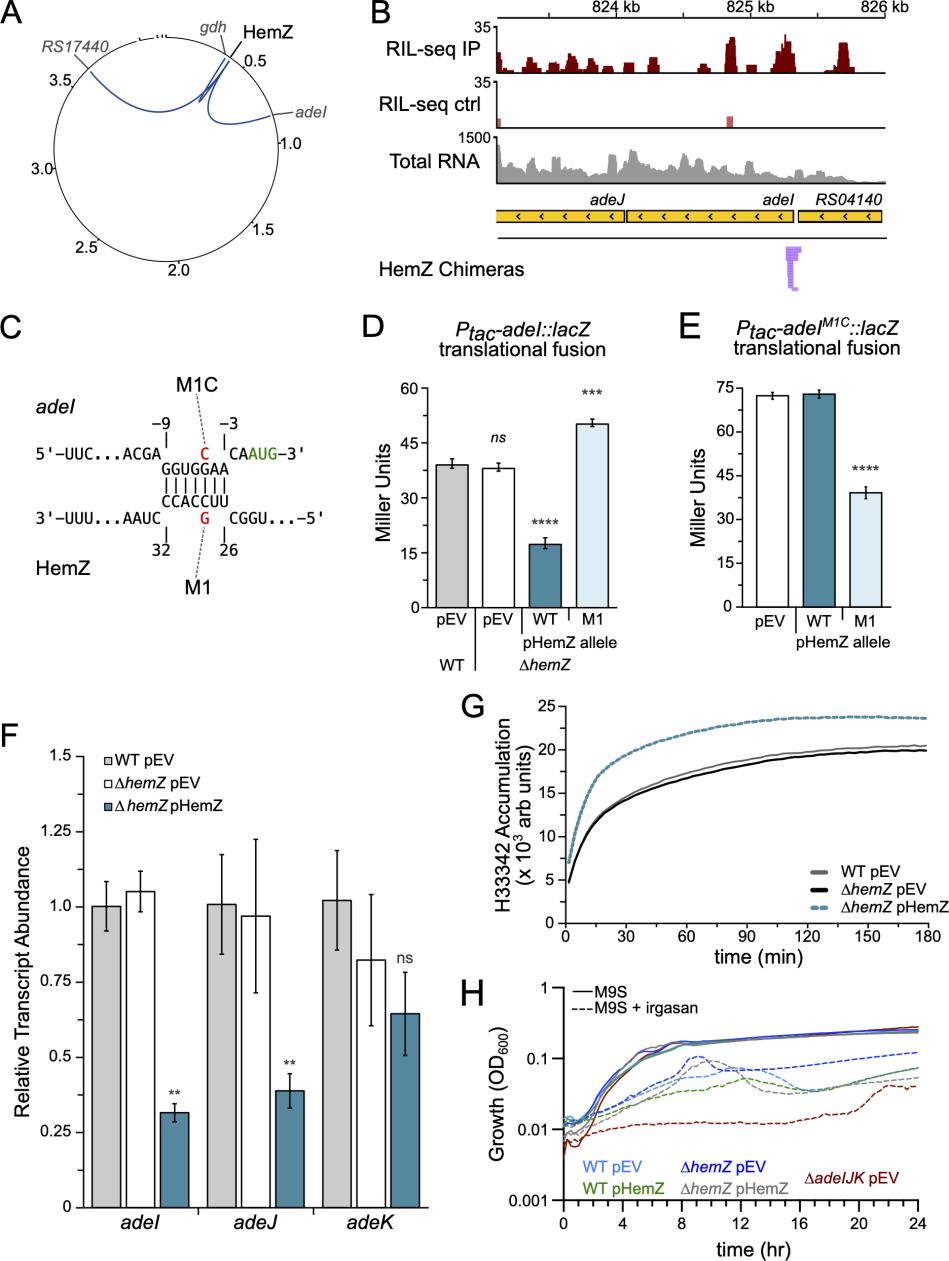
HemZ regulates expression of AdeI. (**A**) Circos (37) plot of RIL-seq chimeras containing HemZ. Circle represents the chromosome and three plasmids of AB5075-UW, numbers indicate genome position (in Megabases). HemZ location is indicated in black text, gray text indicates RNA targets. Links between HemZ and other positions indicate chimeras detected in exponential phase. (**B**) RIL-seq data for *adeI* region. RIL-seq IP panel indicates read depth detected in a representative exponential phase IP sample. RIL-seq ctrl panel indicates read depth detected in a representative exponential phase control sample. Total RNA panel indicates read depth in a representative total RNAseq sample. HemZ chimeras panel indicates position of chimeras detected containing HemZ as RNA2. Yellow bars indicate annotations. The internal black arrows indicate the direction of transcription. For IP, control, and total RNA tracks, only reads mapping to the reverse strand are shown. (**C**) IntaRNA (38) prediction of the interaction between *adeI* and HemZ. The *adeI* start codon is indicated in green text. Locations of the HemZ-M1 and *adeI-M1C* mutant alleles are indicated. Numbers indicate the nucleotide position relative to the start codon of *basJ* (above) and first nucleotide of HemZ (below). (**D**) β-galactosidase activity (in Miller Units) of AB5075 WT cells or its ∆*hemZ* derivative containing a translational *adeI::lacZ* reporter under control of an IPTG-inducible promoter (P*_tac_*) integrated at the Tn*7* attachment site. For these experiments, cells were grown in the presence of 0.5 mM IPTG and 50 ng/mL aTc to induce expression of the reporter and the sRNAs harbored on the indicated sRNA expression plasmids: empty vector control (pEV), wild-type HemZ (pHemZ-WT), or HemZ-M1 mutant allele (pHemZ-M1). Cells were grown for 4 h prior to assessing β-galactosidase activity. (**E**) β-galactosidase activity (in Miller Units) of AB5075 ∆*hemZ* cells containing a translational *adeI^M1C^::lacZ* reporter under control of an IPTG-inducible promoter (P*_tac_*) integrated at the Tn*7* attachment site. Cells were grown as shown in panel D. β-galactosidase assays in panels D and E were repeated independently at least twice with biological triplicate cultures. Data from a single representative experiment are plotted as the mean activity with error bars representing one SD of the mean. (**F**) qRT-PCR analysis of *adeI*, *adeJ*, and *adeK* relative transcript abundance in WT AB5075 cells harboring the empty vector (pEV, gray bars), ∆*hemZ* cells harboring the empty vector (pEV, white bars), and *∆hemZ* cells harboring the HemZ expression vector (pHemZ, blue bars). Total RNA was extracted from biological triplicate cultures that had been grown in LB with inducer (50 ng/mL aTc) for 3.5 h (OD_600_ ≈ 0.6). The RNA was treated with DNase, converted to cDNA, and qRT-PCR was performed. Data are plotted as the relative transcript abundance for the indicated genes compared to the *recA* transcript using the comparative Ct method (2^–∆∆CT^). qRT-PCR experiment was independently repeated twice and data shown from a representative experiment. (**G**) H33342 accumulation assay. Exponentially growing cells (OD_600_ ≈ 0.6) of WT AB5075 with empty vector (pEV; gray line), ∆*hemZ* with empty vector (∆*hemZ* pEV, black line), or ∆*hemZ* with HemZ expression vector (∆*hemZ* pHemZ; dashed blue line) were washed in PBS and mixed with H33342 dye at a final concentration of 25 µM in a 96-well dish in technical quadruplicate. The assay plates were monitored for H33342 fluorescence (excitation: 355 nm, emission: 460 nm) in a microplate reader with readings every 3 min for a total of 3 h at ambient temperature. Data is displayed as the mean fluorescence value for each strain/plasmid condition. Cultures included 50 ng/mL aTc to induce expression from P*_tetA_* on the vector. The experiment was repeated independently three times with a single, representative experiment run displayed. (**H**) Growth curve in the presence or absence of irgasan. Overnight cultures (in M9-succinate) of indicated strains and plasmids were refreshed into M9-succinate containing 5 ng/mL aTc with or without 250 nM irgasan in triplicate wells of a 96-well plate. Growth was monitored every 15 min at OD_600_ in a SpectraMax 190 plate reader at 37°C for 24 h. Data plotted as the mean OD_600_ from the three wells; error bars are omitted for clarity. The assay was repeated independently three times, and results from a single representative run are shown. For β-galactosidase assays, significance was assessed by two-tailed *t*-tests with multiple comparisons corrections as needed. Asterisks indicate significant differences with *, *P* ≤ 0.05; **, *P*-value ≤ 0.01; ***, *P*-value ≤ 0.001; ****, *P*-value ≤ 0.0001.

One mechanism by which *A. baumannii* develops resistance to antibiotics is through altering the expression of antibiotic efflux pumps, including the resistance-nodulation-cell division (RND) pump encoded by the *adeIJK* genes (47, 48). These three genes form an operon that is regulated by AdeN, a TetR-family transcription regulator (49). AdeN acts as a repressor for the *adeIJK* transcription, and several reports describe MDR clinical isolates harboring disruptions in *adeN* (50, 51). Other studies show that the AdeIJK RND pump is essential for *A. baumannii* survival in various virulence models and for full resistance to multiple classes of antibiotics (31, 32, 49). In our RIL-seq study, we identified a single interaction between the *adeI* transcript and the 3′-UTR of an mRNA transcript for *hemF*, an essential gene that is predicted to encode a coproporphyrinogen oxidase involved in heme group biosynthesis. A closer examination of the RIL-seq data indicates that a previously undescribed sRNA may be derived from the 3′-UTR of the *hemF* transcript (Fig. 6). We have named this novel sRNA HemZ. The *adeI*-HemZ interaction was detected in the exponential phase data set. HemZ also formed chimeric interactions with two additional RNA transcripts in the exponential phase RIL-seq experiment: the 5′-UTR of *ABUW_RS01715* (old locus tag: *ABUW_0350*), which encodes a predicted glutamate dehydrogenase enzyme (hereafter referred to as *gdh*), and *ABUW_RS17440* (old locus tag: *ABUW_3583*), which encodes an OmpW-family porin protein (Fig. 6A).

The abundance of HemZ increases as cells enter stationary phase, and HemZ also requires Hfq for its stability, as cells depleted of Hfq show decreased HemZ levels (Fig. 3). Northern blotting indicates that HemZ is a relatively short sRNA (less than 50 nt), and despite multiple attempts to map the 5′ end of HemZ using 5′ RACE, we were unable to definitively identify the start position. We speculate that our RACE experiment may have been inconclusive because multiple forms of HemZ exist (Fig. 3; Fig. S7). Despite our inability to precisely determine whether HemZ is processed from the longer *hemF* transcript or transcribed independently, we created a HemZ expression construct consisting of the last 98 nucleotides of the *hemF* coding sequence and an additional 130 nucleotides downstream of the *hemF* stop codon (Fig. S7). Importantly, inducing expression from the plasmid-borne HemZ construct gives rise to an RNA that reacts with the HemZ northern blot probe and migrates at the same apparent size as the native HemZ sRNA produced in wild-type cells. This suggests that the expression construct includes either the native HemZ-processing signals or a promoter that drives transcription of HemZ (Fig. S7 ).

The AdeI-HemZ chimeras mapped near the predicted translation start site for *adeI*, suggesting that HemZ may regulate the translation of AdeI (Fig. 6B). This hypothesis was corroborated by the IntaRNA algorithm, which predicts that HemZ may base pair with several nucleotides immediately upstream of the AdeI start codon (Fig. 6C). To assess HemZ regulation of AdeI, we generated a translational fusion between *adeI* and *lacZ* comprising the first 15 amino acid residues of AdeI. The *adeI::lacZ* fusion was cloned downstream of an IPTG-inducible promoter (P*_tac_*) and introduced into the Tn*7*-attachment site in wild-type AB5075 and an isogenic derivative thereof lacking *hemZ* (∆*hemZ*). As a control, we first determined whether ectopic expression of HemZ affected the activity of the P*_tac_* promoter and, importantly, observed no significant differences in reporter activity between cells lacking or overproducing HemZ (Fig. S7 ). When we then tested the translational *adeI::lacZ* fusion, we observed that ∆*hemZ* cells harboring plasmid pHemZ produced significantly less reporter activity upon ectopic expression of the HemZ sRNA. We also assessed reporter activity in cells expressing a mutant allele of HemZ containing a single C to G mutation in the predicted HemZ-*adeI* interaction site (HemZ-M1) and observed that the *adeI::lacZ* reporter activity was no longer reduced (Fig. 6D). By introducing a compensatory G to C mutation in the *adeI* sequence (*adeI*^M1C^), the mutant HemZ-M1 allele regained the ability to repress the reporter activity (Fig. 6E). Taken together, these results indicate that HemZ represses the translation of *adeI* through a direct interaction near the translation initiation region of the *adeI* reading frame.

### Regulation by HemZ impacts AdeIJK efflux activity

Since *adeI* is the first gene in the *adeIJK* transcript, we next sought to determine how HemZ’s effect on *adeI* translation impacts the expression of the longer *adeI-adeJK* transcript. To do so, we measured the transcript abundance for *adeI*, *adeJ*, and *adeK* using quantitative reverse transcriptase PCR (qRT-PCR). Consistent with the beta-galactosidase data shown in Fig. 6C, cells lacking *hemZ* did not display significantly altered mRNA abundance for *adeI*, *adeJ*, or *adeK* (Fig. 5F). We did, however, observe that ectopic expression of HemZ resulted in a significant reduction in transcript abundance for both *adeI* and *adeJ*. While the signal for *adeK* trended lower in cells expressing HemZ, the difference did not reach statistical significance. We conclude that HemZ pairs directly with the *adeIJK* mRNA near the translation initiation region for *adeI,* and the effect of this interaction results in decreased abundance of the *adeIJK* transcript.

Having demonstrated that HemZ directly interacts with the *adeIJK* transcript, and given that the AdeIJK proteins constitute an RND transporter, we next wondered if HemZ levels impact drug efflux activity in *A. baumannii*. To explore this possibility, we monitored the accumulation of a dye, Hoechst 33342 (H33342), that fluoresces in the presence of DNA (52, 53). Importantly, H33342 is a known AdeIJK substrate (54). In this assay, the rate of dye accumulation is inversely proportional to the efflux capacity of the cell (i.e., increased dye accumulation indicates decreased efflux activity). As controls, we assayed H33342 accumulation in heat-killed cells and in cells treated with carbonyl cyanide-m-chlorophenyl hydrazone (CCCP), which impairs efflux pump activity by depolarizing the bacterial membrane. In both cases, the rate of H33342 accumulation increased relative to untreated cells, as anticipated (Fig. S8). As an additional validation for the assay, we also measured efflux capacity in cells harboring a deletion of *adeN* (∆*adeN*), a negative regulator of *adeIJK* transcription, and in cells wherein the entire *adeIJK* transcript had been deleted (∆*adeIJK*). These control strains behaved as expected. We observed decreased H33342 accumulation in the ∆*adeN* strain background compared to WT AB5075 and increased accumulation in the ∆*adeIJK* background (Fig. S8). We then performed the assay on either WT or ∆*hemZ* cells expressing HemZ from a plasmid and found that ectopic expression of HemZ led to increased accumulation of the H33342 dye, suggesting a decrease in efflux capacity in response to elevated HemZ levels (Fig. 6G). Importantly, ectopic expression of HemZ had no impact on efflux capacity in the ∆*adeIJK* strain background (Fig. S8), indicating that the observed changes are specific to the HemZ-*adeI* interaction.

We next investigated whether HemZ’s regulation over the *adeIJK* efflux pump impacts antimicrobial sensitivities. The AdeIJK pump contributes to the efflux of various antimicrobial compounds (55). In addition to AdeIJK, *A. baumannii* genomes encode for other RND-type efflux pumps, including both AdeABC and AdeFGH. It has been shown previously that the Ade pumps have overlapping efflux substrate specificities (48). Interestingly, however, the AdeIJK pump appears to be required for resistance to irgasan—also known as triclosan, a common ingredient in antibacterial hand soaps (Fig. 6H) (31). When grown in the presence of sub-inhibitory concentrations of irgasan, we observed a slight increase in growth yield for cells lacking *hemZ* compared to wild-type cells. Cells lacking *adeIJK* grew very poorly in the presence of irgasan. On the other hand, ectopic expression of HemZ from a plasmid in the presence of sub-inhibitory concentrations of irgasan led to decreased growth in both WT and *∆hemZ* cells (Fig. 6H). Collectively, our investigations suggest that HemZ negatively regulates the expression of *adeI* through a direct interaction with the *adeI* translation initiation region. This regulation impacts the activity of the AdeIJK efflux pump and alters drug efflux capacity. The regulatory effects of HemZ on AdeIJK further impact the susceptibility of *A. baumannii* to irgasan, an ingredient often used in antibacterial hand soaps.

## DISCUSSION

Here, we experimentally determined the RNA-RNA interactome mediated by Hfq in *A. baumannii* using RIL-seq. Consistent with the role of Hfq in other organisms, this key post-transcriptional regulator coordinates a robust network of RNA-RNA interactions in *A. baumannii*. We detected over 1,100 chimeric RNAs encompassing 98 known and previously undescribed sRNAs. Our RIL-seq data sets further indicate that Hfq likely facilitates the regulatory effects for numerous sRNAs, many of which are newly described sRNAs that appear to be derived from the 3′-UTR of longer transcripts, including PtaZ and HemZ, which regulate clinically relevant aspects of *A. baumannii* physiology. Taken together, our RIL-seq experiments provide a comprehensive look at the post-transcriptional regulatory network in this troublesome bacterial pathogen.

Previous studies identified *hfq* as a candidate essential gene in *A. baumannii* strain AB5075 (7, 8). In the antibiotic-susceptible *A. baumannii* ATCC 17978 strain background, *hfq* is not essential. Mutant derivatives lacking *hfq* display pleiotropic phenotypes, including increased sensitivity to environmental stressors, impaired carbon source utilization, and defects in virulence models (10–12). Using CRISPRi, we found that depletion of *hfq* in several *A. baumannii* isolates inhibited growth (Fig. 1). An exception to this observation was strain ATCC 17978, wherein *hfq* depletion did not fully abrogate growth. Taken together, the CRISPRi data and the aforementioned studies strongly suggest that Hfq plays a key role in regulating the physiology of *A. baumannii*. The results of our RIL-seq experiment lend further support to this hypothesis, and our data are consistent with the idea that Hfq orchestrates a dynamic network of post-transcriptional regulation in this important pathogen. Indeed, RIL-seq indicates that Hfq associates with numerous transcripts spanning pathways known to be important for *A. baumannii* physiology, including metabolism, nutrient acquisition, transcription, and translation (Table 1). In addition, when we cross-referenced TnSeq data sets collected for strain AB5075, we found that Hfq interacts with numerous transcripts encoding essential genes and genes required for both virulence and antibiotic resistance (Table S1). As the major concern with *A. baumannii* infections remains the continued emergence of antibiotic resistance in currently circulating strains, we were initially interested in uncovering novel post-transcriptional regulators, namely Hfq-interacting sRNAs, that may influence the expression of transcripts related to virulence and antibiotic resistance. We chose three sRNAs for our initial follow-up experiments: PtaZ, HemZ, and Aar.

We found that PtaZ, a previously undescribed sRNA that may be generated from the longer *ackA-pta* transcript, directly regulates the translation of BasJ, an isochorismate synthase enzyme involved with production of the acinetobactin siderophore (Fig. 5) (44). Interestingly, the acinetobactin siderophore, one of several siderophores produced by *A. baumannii* isolates, was recently shown to be critical for virulence in a murine infection model (43). We were initially interested in exploring the regulatory role of PtaZ because it formed chimeras with the mRNA encoding Oxa23, a major determinant of carbapenem resistance in *A. baumannii* (39). While we detected PtaZ-Oxa23 chimeric RNAs in both exponential and stationary phase data sets, we have not yet observed regulatory effects of PtaZ over Oxa23, either in terms of protein levels or the antibiotic resistance profile in cells lacking (∆*ptaZ*) or overexpressing PtaZ (data not shown). The PtaZ-*oxa23* chimeras were positioned in the latter third of the *oxa23* open reading frame, suggesting that PtaZ may not impact the translation of Oxa23. Our 5′-RACE results indicate that the PtaZ sRNA starts at a position near the end of the *ackA-pta* transcript. Whether PtaZ is derived from RNase processing of the longer *ackA-pta* transcript or is transcribed from its own promoter is currently unclear. The *ack-pta* transcript encodes for acetate kinase (*ackA*) and phosphate acetyltransferase, respectively. In *E. coli*, AckA and Pta are key players in acetate dissimilation—a process that links energy metabolism with carbon and phosphorus levels via overflow metabolism (56). It is intriguing, then, to speculate that in *A. baumannii*, the regulatory effects of PtaZ may help fine-tune gene expression during changing growth conditions. Consistent with this possibility, PtaZ formed chimeras with additional transcripts encoding putative metabolite transport proteins, including *cstA* (carbon starvation protein A, *ABUW_RS05590*; old locus tag: *ABUW_1142*), a predicted pyruvate transporter, and *ABUW_RS06645* (old locus tag: *ABUW*_1362), which encodes a predicted dicarboxylate or amino acid transporter.

In addition to horizontally acquired antibiotic resistance mechanisms, *A. baumannii* genomes also encode numerous intrinsic resistance factors, including antibiotic efflux pumps. In our RIL-seq data, we detected chimeras with *adeI*, the first gene in the *adeIJK* transcript that encodes for the AdeIJK RND-type efflux pump. The AdeIJK pump is highly conserved across the *A. baumannii* species, and the pump plays a role in exporting a wide variety of antibiotics (47, 50). In addition to mediating antibiotic resistance, the AdeIJK efflux pump was previously identified as a candidate virulence gene in the *Galleria mellonella* virulence model, suggesting that the role of AdeIJK may extend beyond merely exporting antibiotics (32). Here, we showed that HemZ, a novel 3′-UTR-derived sRNA, directly regulates the translation of *adeI*, thereby decreasing the efflux capacity of *A. baumannii* AB5075 cells and impacting resistance to irgasan, a commonly used antimicrobial (Fig. 6G and H). We further showed that HemZ causes a reduction in mRNA transcript abundance for both *adeI* and *adeJ* (Fig. 6F). We speculate that the interaction of HemZ with the *adeI* mRNA uncouples transcription and translation of the *adeIJK* transcript, leading to decreased processivity of RNA polymerase when transcribing this *adeIJK* operon. However, the lack of a significant reduction in *adeK* transcript abundance suggests that other factors may be at play. Possible explanations for this discrepancy include RNA processing events that differentially affect the longer *adeIJK* transcript in the context of HemZ activity, or the presence of an alternative promoter driving transcription of *adeK* independently of the longer *adeIJK* transcript. Our data further suggest that HemZ, which regulates the *adeIJK* transcript, may be processed from the longer *hemF* transcript (Fig. S7). HemF is a candidate essential gene in *A. baumannii* (7, 8) and encodes for an oxygen-dependent coproporphyrinogen oxidase that is predicted to be involved in heme group biosynthesis. In addition to targeting *adeI*, HemZ also formed a chimeric pairing with the 5′-untranslated region of an mRNA of *gdh*, which encodes a predicted glutamate dehydrogenase enzyme (Fig. 6). Because glutamate is a key precursor in the heme/porphyrin biosynthetic pathway, an intriguing hypothesis is that by regulating *gdh* expression, HemZ may function to regulate glutamate levels to coordinate heme group biosynthesis.

In our RIL-seq data, Aar was detected as a predominant Hfq-associated sRNA (Fig. 2) and formed chimeras with 29 different RNAs: 12 in the exponential phase, 21 in stationary phase with 4 targets detected in both data sets. In the recent Hi-GRIL-seq RNA-RNA interactome study by Hamrock and colleagues, Aar was similarly identified as a prominent sRNA and interacted with 98 distinct RNA transcripts (17). There was limited overlap between the Aar regulatory targets detected by Hamrock et al. and our RIL-seq data; only seven RNA transcripts were found to interact with Aar by both experimental methods. Several possible explanations for these differences include the growth conditions used for each experiment, technical differences in the sequencing library construction, or the differential sensitivity/specificity of the two approaches. For example, in the Hi-GRIL-seq data, 18 of the 98 Aar interaction partners are tRNAs. We did not detect any Aar-tRNA interaction pairs occurring on Hfq in our experiments. This could mean that interactions between Aar and certain tRNAs may not be mediated by an RNA chaperone or might be mediated by an RNA chaperone other than Hfq. It is interesting to note that a similarly limited overlap was observed when comparing Hi-GRIL-seq results with Hfq RIL-seq results in *P. aeruginosa* (19, 57), which might suggest that technical differences between the two experimental approaches may be a leading cause.

The data presented herein strengthens the previous findings that Aar regulates *carO* and indicates that Aar’s regulatory effects are potentially mediated by Hfq. We demonstrated that Aar directly interacts with the *carO* transcript in the multidrug-resistant AB5075 strain and further regulates CarO abundance in a non-carbapenem-resistant strain *A. baumannii* ATCC 17978 (Fig. 4), suggesting that Aar’s ability to repress CarO expression is not strain-specific and that this regulatory relationship may be conserved across the species. Previous reports have implicated that carbapenem-resistant clinical isolates of *A. baumannii* often display altered or absent CarO expression, leading multiple groups to speculate that CarO may be a transporter for carbapenem antibiotics (35). *In vitro*, recombinant CarO allows transport of basic amino acids, such as glycine and ornithine, and possibly imipenem (58, 59). We thus explored the possibility that the regulatory effects of Aar on CarO may lead to altered carbapenem resistance. This was not the case, and in our hands, CarO had no discernible role for carbapenem resistance for either strain AB5075 or 17978 (Table 2). For AB5075, this observation has been attributed to the presence of a highly active carbapenemase, Oxa23, in the strain (39). Contrary to this hypothesis, deletion of *carO* did not alter carbapenem resistance in an AB5075 background lacking *oxa23* (Table 2). These data indicate that *A. baumannii* likely harbors one or more additional porin proteins that allow for the uptake of carbapenems.

If *carO* is not involved with carbapenem resistance directly, what might explain the observations among clinical isolates showing mutations or decreased expression of CarO? One possibility stems from recent studies which demonstrate that CarO serves as a receptor for bacteriophage (40, 60). It is thus possible that clinical isolates exhibit decreased CarO levels due to pressure from phage predation, in addition to escaping antibiotic pressure. As noted above, Aar interacts with and likely exerts regulatory control over several other transcripts. These additional targets include *murC*, which is involved in the early stages of peptidoglycan biosynthesis, as well as multiple enzymes involved with nucleic acid modifications, such as transcripts for two ribosomal RNA modification enzymes, *rrm* and *rsuA*, and *ung*, which encodes an uracil-DNA glycosylase involved with DNA repair. Here, we demonstrated that Aar regulates the expression of an *rsuA::lacZ* translational reporter (Fig. 4). Based on these observations, an intriguing possibility is that Aar is produced in response to phage predation and subsequently targets multiple pathways relevant to phage defense, including potential outer membrane receptors (*bfnH*, *carO*), cell wall homeostasis (*murC*), and nucleic acid modification pathways (*ung*, *rrm*, *rsuA*). It will thus be important to determine if Aar plays a role in mediating resistance to phage attack through its regulatory targets.

The RIL-seq approach applied here specifically detects RNA-RNA interactions that occur on Hfq. Through this approach, we detected RNA interaction partners for 98 different sRNAs, 60 of which have been previously described. In the recent Hi-GRIL-seq study, Hamrock and colleagues detected interaction partners for 40 sRNAs in *A. baumannii* AB5075 (17). We detected 24 of these sRNAs in our RIL-seq experiment. This extensive overlap suggests that Hfq may be involved in facilitating the regulatory activities for many of the sRNA-mRNA pairs detected using the Hi-GRIL-seq approach. In addition to detecting overlap with many sRNAs identified in the Hi-GRIL-seq study, our RIL-seq approach also revealed RNA interaction partners for an additional 74 sRNAs. Of these, 38 sRNAs have not been previously described (Table S2), including two of the sRNAs we investigated here: PtaZ and HemZ. Taken together, our data provide a snapshot of the extensive network of sRNAs—and regulatory targets thereof—that are orchestrated by Hfq in *A. baumannii*.

To conclude, we examined the repertoire of RNA-RNA interactions mediated by Hfq in *A. baumannii*. Similar to the results from RIL-seq experiments conducted in other bacterial species, our data suggest that Hfq functions as a global post-transcriptional regulator in *A. baumannii* and facilitates interactions between hundreds of RNA transcripts. Our data further provides a comprehensive look at the Hfq-mediated post-transcriptional regulatory network in this troublesome opportunistic pathogen. Importantly, we identified RNA targets for nearly 100 sRNAs, including numerous sRNAs that have not been described previously. As a number of these newly described sRNAs appear to be derived from the 3′-untranslated regions of longer RNA transcripts, it will be important to explore the RNA processing events which lead to the production of these regulatory RNAs and how those activities further influence post-transcriptional regulation.

## MATERIALS AND METHODS

### Bacterial strains and growth conditions

Bacterial strains are listed in Table S3. *Acinetobacter baumannii* strains were routinely grown in lysogeny broth (LB, 10 g/L NaCl, 10 g/L tryptone, 5 g/L yeast extract) at 37°C with shaking at 200 rpm, or on LB agar plates solidified with Difco Bactoagar (15 g/L; BD, cat. no. 214010). For experiments conducted in minimal medium, cells were grown in 1× M9 salts (6 g/L Na_2_HPO_4_, 3 g/L KH_2_P0_4_, 0.5 g/L NaCl, 1 g/L NH_4_Cl, 0.2 mM CaCl_2_, 1 mM MgSO_4_) with succinate (20 mM) as the sole carbon source. Antibiotics were added to growth media as needed: apramycin, 50 µg/mL (solid), 25 µg/mL (liquid); hygromycin, 250 µg/mL; chloramphenicol, 5 µg/mL; tetracycline, 10 µg/mL; kanamycin, 50 µg/mL (solid), 25 µg/mL (liquid); and carbenicillin, 100 µg/mL (solid), 50 µg/mL (liquid). Anhydrotetracycline (aTc, Fisher Scientific, cat. no. 10009542) was dissolved in DMSO at a stock concentration of 1 mg/mL and diluted to the final working concentrations as needed. As a vehicle control, an equivalent dilution of DMSO was performed. Isopropyl-beta-D-thiogalactopyranoside (IPTG, Research Products International, cat. no. I56000) was prepared at a stock concentration of 1 M and diluted to the final working concentrations as indicated below or in the figure legends.

### Plasmid construction

Oligonucleotide primers, plasmids, and synthetic DNA fragments (gBlocks) can be found in Table S4 to S6, respectively. For cloning purposes, *E. coli* DH5⍺ was used. Routinely, plasmid backbones were digested with restriction enzymes as recommended by the manufacturer (NEB), treated with QuickCIP (NEB, cat. no. M0525S), and purified after agarose gel electrophoresis. PCR products were generated with KOD Extreme Hot Start DNA Polymerase (Sigma Aldrich, cat. no. 71975) in 50 µL reactions, as recommended by the manufacturer. Primers used for cloning additionally contained 20–25 nucleotides of homologous bases at the 5′ ends to facilitate cloning via isothermal assembly (ITA) (61). ITA cloning reactions (10 µL volume) were carried out at 50°C for 20 min. The ITA reaction was subsequently transformed into chemically competent DH5α. All plasmid inserts were verified by Sanger sequencing (Iowa State University DNA Facility) or whole plasmid sequencing (Plasmidsaurus). Full details of plasmid construction can be found in the supplementary methods.

### Preparation and transformation of electrocompetent *A. baumannii*

*A. baumannii* cells were prepared for electroporation as described previously (9). Cells from 1.5 mL of an overnight LB culture were pelleted in microcentrifuge tubes and washed thrice with 0.3 M sucrose (1 mL per wash). After the final wash, 400 µL of 0.3 M sucrose was used to resuspend the cells. For electroporation, 70 µL of cell suspension was mixed with approximately 50–100 ng of plasmid DNA, and electroporation was carried out using setting EC2 on a Bio-Rad Micropulser. Immediately after electroporating, cells were recovered in ≈900 µL LB at 37°C for 60 min with shaking before the cells were plated on the appropriate selective media.

### Introduction of Tn*7* plasmids to *A. baumannii*

Four-parental matings were used to introduce Tn*7*-delivery plasmids into *A. baumannii* strains using established protocols (32). Overnight cultures—including the recipient *A. baumannii* cells, a donor strain of *E. coli* (strain LW264) harboring the Tn*7*-delivery plasmid (donor), and two helper strains, *E. coli* TOP10 pRK2013 and *E. coli* DH5α(λpir) pTNS3—were mixed (0.1 mL of each parent) and pelleted by centrifugation at 7,000 × *g* for 2 min. The resulting pellet was washed with 0.5 mL fresh LB and pelleted again, after which the pellet was suspended in 30 µL of LB and spotted on solid LB agar. The liquid was allowed to dry at room temperature, and plates were incubated at 37°C for 6–8 h. The resulting growth patches were collected in 1 mL of sterile PBS, and transconjugants were selected on LB plates with hygromycin (250 µg/mL) and chloramphenicol (5 µg/mL). Colony PCR was used to verify the proper integration of the Tn*7* cassette.

### Construction of *A. baumannii* mutant strains

*A. baumannii* mutant strains were generated using derivatives of pMJG42, an allele exchange vector that confers resistance to tetracycline and sensitivity to sucrose (32). The pMJG42 derivatives were conjugated into *A. baumannii* cells via donor *E. coli* strain LW264, and transconjugants were selected on LB plates containing tetracycline and chloramphenicol. Primary integrants were purified once and plated on yeast-tryptone plates (5 g/L yeast extract, 10 g/L tryptone, 15 g/L Bacto agar) containing 5 % (wt/vol) sucrose to select for clones that had resolved the pMJG42 plasmid backbone. Sucrose-resistant colonies were screened by colony PCR and/or DNA sequencing to identify clones harboring the desired mutation.

### RIL-seq experimental procedures

#### Collection of RIL-seq samples

RIL-seq was performed as previously described (24). For RIL-seq with Hfq in *A. baumannii*, triplicate overnight cultures of wild-type AB5075 and AB5075 Hfq-V were refreshed to an initial OD_600_ of 0.01 and grown until exponential phase (OD_600_ ≈ 0.6) and early stationary phase (OD_600_ ≈ 2.0). At each time point, the equivalent of 40 OD_600_ units was collected and processed for RIL-seq. Cells were pelleted, washed with PBS, and exposed to ultraviolet radiation to form cross-links between nucleic acids and proteins. At this point, a portion of each sample was retained for total RNA-seq analysis. Following cross-linking, an immunoprecipitation (IP) was performed with murine monoclonal anti-VSV-G antibodies (Sigma Aldrich, cat. no. SAB4200695). After the IP, RNA ends were trimmed using an RNase cocktail (RNaseA/T1, Thermo Fisher Scientific, cat. no EN0551), phosphorylated by T4 polynucleotide kinase (NEB, cat. no. M0201), ligated by T4 RNA Ligase 1 (NEB, cat. no. M0437M), and treated with proteinase K (Thermo Fisher Scientific, cat. no. EO0491). RNA was extracted using TriReagent LS (Sigma-Aldrich, cat. no. T9424) according to the manufacturer’s recommendations. Sequencing libraries for the RIL-seq samples were prepared for Illumina sequencing using the RNAtag-seq protocol (62) with adaptations to capture bacterial sRNAs (24). The Ribo-Zero Plus rRNA depletion kit (Illumina, cat. no. 20040526) was used to remove rRNAs. The RIL-seq libraries were sequenced at the Harvard Medical School Biopolymers Facility by paired-end sequencing on a NextSeq500 Sequencer (Illumina). Throughout the RIL-seq library construction, RNA/cDNA quality was assessed by the Molecular Genetics Core Facility at Boston Children’s Hospital. The total RNA samples were prepared and sequenced by SeqCenter. Briefly, the total RNA samples were treated with RNAse-free DNAse (Invitrogen) prior to library preparation using Illumina’s Stranded Total RNA Prep Ligation with Ribo-Zero Plus kit and 10 bp IDT for Illumina indices. The libraries were sequenced on a NextSeq 2000, giving 2 × 50 bp reads. Demultiplexing, quality control, and adapter trimming were performed with Illumina software bcl-convert (v3.9.3).

#### Computational analysis of RIL-seq

The analyses of RIL-seq were carried out as previously described (19, 24), with modifications to the pipeline for the *A. baumannii* AB5075-UW genome and plasmids (NCBI RefSeq Assembly GCF_000963815.1). The RIL-seq software distribution (version 0.78) was downloaded at https://github.com/asafpr/RILseq. The raw sequencing reads were processed to remove adapter sequences, as well as reads with low complexity and low quality, using Cutadapt version 2.10 (63). The first 25 bases of each read were then mapped to the AB5075-UW genome and plasmids using the RIL-seq script ‘map_single_fragments.py’ program, which uses the bwa alignment program (64) in paired-end mode, allowing for 2 mismatches and a maximum insert size of 1,500 bp. Fragments were then classified as “single” or “chimeric,” with chimeric reads defined as those where each individual read maps to different strands, or where the reads map more than 1,000 bp apart on the same strand. Following this step, chimeras were assessed for significance by dividing the genome into non-overlapping 100-nt windows and counting the number of chimeras in each possible pair of windows. The three replicates from each RIL-seq data set (i.e., Hfq-V exponential phase, Hfq-V stationary phase) were assessed and found to be reproducible as previously described (Fig. S2) (20, 24), and the three replicates were merged into a single unified data set. Fisher’s exact test was then applied to determine which chimeric fragments were significantly overrepresented beyond what would be expected at random, based on the number of chimeric and single fragments mapping to each region (*P* ≤ 0.05 following Bonferroni correction). As the RIL-seq analytical pipeline accepts any chimera identified five or more times by default, we applied more stringent cutoff values to only accept those chimeras that were detected at a frequency higher than 90% of the chimeras detected in the control condition (i.e., wild-type AB5075) for each RIL-seq data set (i.e., exponential and stationary phase). To satisfy this more stringent cutoff value, we accepted chimeras detected at least 13 and 11 times for the exponential and stationary phase data sets, respectively (Data set S1 ). For the resulting chimeras, we assigned each read to its corresponding gene in the AB5075-UW genome and removed any chimeras that may originate from a single transcript but at positions more than 1,000 bp apart. These potentially self-derived chimeras are included in Data set S1. An IGV-web browser session (65) was generated for both the exponential phase data set (link: https://tinyurl.com/4xa9y3y2) and the stationary phase data set (link: https://tinyurl.com/4ae3d4y4). For the total RNA RNA-seq samples, the reads were mapped to the AB5075-UW genome using bowtie2 version 2.4.5 (66) with the following settings: -N 1L 15 -i S,1,1.00 -D 20 --non-deterministic --no-mixed --no-discordant --no-unal. The mapped reads were converted to the bedgraph format, normalized to a read depth of 10 million reads per sample using bedtools version 2.30.0 (67), and then converted to the bigwig file format for visualization in IGV (65). Sequencing reads and processed files have been deposited at Gene Expression Omnibus repository under accession numbers GSE295534 (RIL-seq IP samples) and GSE295535 (RIL-seq total RNA samples).

#### Generation of Circos plots and extraction of specific chimeric reads

Circos version 0.69 (37) was used to create the plots depicted in Fig. 4 to 6. For each sRNA, all chimeric interactions containing the sRNA were plotted and colored by data set (i.e., exponential and stationary phase). The “chimeras” tracks in the IGV browser images depicted in Fig. 4 and 6 were generated by first extracting all the chimeric fragments containing the sRNA of interest from the corresponding RIL-seq data set based on the genomic coordinates of the sRNA. The RIL-seq package script ‘generate_BED_file_of_endpoint.py’ was then used to create a BED file of the sequencing reads containing the sRNA of interest, which was subsequently converted to the bam file format for visualization in IGV using bedtools (67).

### CRISPRi

CRISPRi-mediated knockdown of *hfq* expression was conducted in strain backgrounds harboring a defective *cas9* gene from *Streptococcus pyogenes* integrated at the Tn*7* attachment site (7, 9). *A. baumannii* strains harboring the *dcas9* cassette were transformed with single guide RNA (sgRNA) expression plasmids that constitutively expressed a control sgRNA (targeting the *mCherry* gene, which is absent from the AB5075 genome) or an sgRNA targeting the non-template strand of *hfq* or *csrA*. For plate-based assays, individual colonies were suspended in sterile PBS, serially diluted, and 10 µL aliquots of each dilution were plated on LB-apramycin plates with or without 100 ng/mL aTc. The plates were dried at ambient temperature and incubated overnight at 37°C. For liquid-based assays, overnight cultures were refreshed to an initial density of OD_600_ 0.01 and grown to early exponential phase (OD_600_ ≈ 0.4–0.5), at which time dCas9 expression was induced by the addition of 50 ng/mL aTc. For northern blot analyses with the AB5075 *dcas9* strain, RNA samples were collected at time 0 (i.e., immediately prior to induction) and at 1, 2, and 4 h post-induction. RNA was extracted as described below. For Western blot analyses in the Hfq-V *dcas9* strain, cell pellets were collected at time 0 (immediately prior to induction) and at 0.5, 1, 2, 3, and 4 h post-induction and processed as described below.

### Northern blotting

For northern blot analyses, RNA was extracted with TriReagent LS according to the manufacturer’s recommendations. Briefly, cell pellets (equivalent to 10 OD units) were dissolved in 1 mL room temperature TriReagent and incubated at room temperature for 5 min. A 100 µL portion of 1-bromo-2-chloropropane (Sigma Aldrich, cat. no. B9673) was added and mixed thoroughly. After a 10-minute incubation at room temperature, samples were centrifuged for 15 min at full speed in a 4°C tabletop microcentrifuge, and the aqueous layer was transferred to a fresh tube containing 0.5 mL isopropanol to precipitate nucleic acids. The samples were mixed by inversion, incubated at room temperature for 10 min, and centrifuged at full speed for 10 min at 4ºC. The resulting RNA pellets were washed once with freshly prepared 75% ethanol, dried at room temperature for 10–15 min, and hydrated with 70 µL of nuclease-free water and quantified using fluorimetry.

Following extraction, 5 µg of RNA were separated on 8% polyacrylamide gels containing 6 M urea (1:4 mix of UreaGel Complete:UreaGel 8; National Diagnostics, cat. no. EC-838-1LTR) in 1× TBE buffer at 300 V for approximately 75 min. RNA was transferred to Hybond-N + nylon membranes (Amersham, cat. no. RPN303B) at 20 V for 16–20 h in 0.5× TBE at 4°C. After the transfer, the membranes were exposed to UV irradiation to cross-link the RNA to the membrane. Single-strand RNA size standards (RNA low-range ladder, NEB, cat. no. N0364S) were marked using UV-shadowing. Membranes were blocked in Ultrahyb-Oligo Hybridization Buffer (Ambion, cat. no. AM8663) for 2 h at 45°C prior to hybridization with labeled oligonucleotide probes (Table S4). With the exception of the 5S rRNA probe (described below), oligonucleotide probes were 5′ ^32^P-end labeled with 0.3 mCi of γ-^32^P ATP (Perkin Elmer, cat. no. BLU035C010MC) using 10 U of T4 polynucleotide kinase (NEB, cat. no. M0201) at 37°C for 1 h. After labeling, probes were purified via MicroSpin G-50 columns (Cytiva, cat. no. 27-5330-01). For hybridization, 40 pmol of radiolabeled probes were added to the pre-blocked membranes and incubated overnight at 45°C, after which the membranes were washed twice with 2× SSC buffer with 0.1% SDS at room temperature and washed three times with 0.2× SSC buffer with 0.1% SDS at room temperature, except for the second wash, which was performed at 45°C for 25 min. Northern blot images were captured on phosphor-storage screens and imaged with an Azure Sapphire PhosphoImager. As needed, the membranes were stripped with three 10-minute incubations in boiling 0.2% SDS and three 10-minute incubations in boiling water before hybridizing with an additional probe. Hybridization with 5S rRNA probe was performed as described above, except for the oligonucleotide probe, which was synthesized with IRDye 700 at the 5′ end (IDT). To capture the 5S rRNA signal, the membrane was imaged using an Azure Sapphire imager.

### Western blotting

For Western blotting, overnight cultures were back-diluted into 5 mL fresh media (LB or M9-succinate, with or without apramycin or induction with aTc, as needed). For experiments grown in LB (CRISPRi and CarO-V), cultures were set to an initial OD_600_ of 0.01. For experiments grown in M9-succinate (BasJ-V), cultures were set to an initial OD_600_ of 0.05. The refreshed cultures were grown for the indicated times (CRISPRi experiment) or for 6 h (CarO-V and BasJ-V experiments) with shaking at 37°C, at which point 500 µL of cells were pelleted and stored at −80°C until further use. For the BasJ-V wash-out experiment, overnight LB cultures of the indicated strains and plasmids were refreshed into LB with 25 µg/mL apramycin and 50 ng/mL aTc at an initial OD_600_ of 0.01. The cultures (5 mL) were incubated with shaking at 37°C for 3 h, at which time the cultures were pelleted, washed with 1× M9 salts, and resuspended in 5 mL of M9-succinate containing 25 µg/mL apramycin and 5 ng/mL aTc. The cultures were incubated with shaking at 37°C, and 500 µL aliquots were harvested for Western blot analysis samples at the indicated times and stored at −80°C until further use. For an iron-replete control condition, 30 µM FeCl_3_ was added.

The frozen cell pellets were thawed on ice and resuspended in 1× NuPAGE LDS sample buffer (ThermoFisher, cat. no. NP0007). Samples were then boiled at 100°C for 10 min, sonicated briefly, and cellular debris was pelleted by centrifugation. Samples were then resolved through a 4–12% NuPAGE Bis-Tris gel (ThermoFisher, cat. no. NP0321) in 1× MOPS SDS running buffer (ThermoFisher, cat. no. NP0001). The gel was transferred to an Immobilon PVDF membrane (EMD Millipore, cat. no. ISEQ08100) in 1× NuPAGE transfer buffer (ThermoFisher, cat. no. NP00061) with 10% methanol and blocked overnight in Li-Cor Intercept blocking buffer (Li-Cor, cat. no. 927-70001) diluted 1:5 in 1× PBS. Primary antibodies were added for 1 h in blocking buffer: rabbit anti-VSVG at 1:3,333 dilution (Sigma Aldrich, cat. no. V4888) and mouse anti-*E*. *coli* RpoA at 1:10,000 dilution (Biolegend, cat. no. WP003). The membrane was washed with 1× PBS + 0.05% Tween for a total of three 10-minute washes, followed by a single 10-minute wash in blocking buffer. IRDye-conjugated secondary antibodies were added for 1 h in 1× PBS + 0.05% Tween: donkey anti-rabbit IgG conjugated with IRDye 800CW (Li-Cor, cat. no. 925-32213) at a 1:10,000 dilution and goat anti-mouse IgG conjugated with the IRDye 680RD (Li-Cor, cat. no. 925-68070) at a 1:30,000 dilution. The membrane was washed for 10 min four times with 1× PBS + 0.05% Tween, followed by two additional 10-minute washes with 1× PBS. After being dehydrated in methanol and allowed to dry at ambient temperature, membranes were imaged on an Azure Sapphire imager using NIR settings (800 CW and 680RD) with 100 µm resolution. Western blot experiments were conducted with biological triplicate samples, repeated independently at least twice, with results depicting a representative experiment run.

### Beta-galactosidase assays

Beta-galactosidase assays were conducted essentially as described previously (9). The *lacZ* reporter fusions were introduced into the bacterial chromosome at the Tn*7*-attachment site, as described above, for wild-type AB5075 or sRNA-deletion derivatives. The reporter strains were electroporated with the indicated plasmids as described above. Assays were conducted with biological triplicate cultures, which were grown overnight in 3 mL LB cultures, with the exception of experiments testing *basJ* reporter constructs, which were cultured in M9-succinate medium. The overnight cultures were refreshed to an OD_600_ of approximately 0.01 (LB experiments) or 0.05 (M9-succinate experiments) in 3 mL of fresh media. Antibiotics were added for plasmid maintenance as needed. When indicated, sRNA expression was induced with 5 ng/mL aTc (M9-succinate) or 50 ng/mL aTc (LB). An equivalent volume of DMSO was used as a vehicle control. For the P*_tac_-adeI::lacZ* reporters, IPTG was added at a concentration of 0.5 mM to induce expression from the P*_tac_* promoter controlling transcription of the *adeI::lacZ* fusions. The refreshed cultures were grown with shaking at 37°C for 6 h (*rsuA* and *basJ* reporters) or 4 h (*adeI* reporters), after which time the cultures were placed on ice for at least 20 min. To assess beta-galactosidase activity, 200 µL of cells were added to 800 µL of Z-buffer (16.1 g/L Na_2_HPO_4_, 5.5 g/L NaH_2_PO_4_, 0.75 g/L KCl, 0.246 g/L MgSO_4_, pH 7.0) + 2.72 µL/mL beta-mercaptoethanol in technical duplicate. The cells were permeabilized with 30 µL of 0.1% SDS and 60 µL of chloroform, and beta-galactosidase activity was measured using ortho-nitrophenyl-β-D-galactopyranoside (ONPG). Reactions were quenched by the addition of 0.5 mL 1M Na_2_CO_3_. Miller Units were calculated using the OD_420_ and OD_550_ values. Data are reported as the mean activity (in Miller Units) from the biological triplicate cultures, with error bars representing one standard deviation. Statistical analyses were performed by two-tailed *t*-tests. All beta-galactosidase experiments were independently repeated at least two times with biological triplicate samples, and the results shown are from a single, representative experiment run.

### Disc diffusion assays

Disc diffusion assays were performed essentially as described previously (9). Briefly, 50 µL of overnight cultures (n = 3 per strain) of the indicated strains were mixed with 4 mL molten LB-top agar (LB media with 0.75 g/L bacto agar), poured onto LB agar plates (25 mL of LB agar) and allowed to solidify at room temperature. Antibiotic-laden filter paper discs were placed on top of the agar. The plates were incubated at 37˚C overnight (≈ 16–18 h), at which point zones of inhibition were measured. Discs contained the following amounts of antibiotic: imipenem (BD, 231644): 10 ng, meropenem (BD, 231703): 10 ng, ceftazidime (BD, 231633): 30 ng; gentamicin (Sigma, G1397): 250 ng; tetracycline (Fisher Scientific BP912): 25 ng. The disc diffusion assay experiment was completed independently three times; data in Table 2 depict the average zone of inhibition from a single representative experiment run.

### 5′ rapid amplification of cDNA ends (RACE)

The 5′ end of the PtaZ sRNA was determined via 5′-RACE using the template switching RT enzyme mix (NEB, M0466) according to the manufacturer’s recommendations. RNA was extracted as described above from 2 mL of culture of the *∆ptaZ* mutant harboring the pPtaZ expression plasmid following 3.5 h of growth in LB with 5 ng/mL aTc to induce expression from the tetracycline-responsive promoter on the plasmid. cDNA was generated using PtaZ-specific oligo PtaZ_RACE and template-switching oligo TSO_RT. The resulting cDNA was amplified using Q5 Hot Start High-Fidelity Master Mix (2×) (NEB, cat. no. M0494) with primers PtaZ_RACE and TSO_NESTED. The resulting PCR reaction was cleaned up using Exo-CIP Rapid PCR Cleanup Kit (NEB, cat. no. #E1050) and sequenced by the Iowa State University DNA Facility.

### Quantitative reverse transcriptase PCR (qRT-PCR)

qRT-PCR was performed using cDNA generated from RNA isolated from the indicated strains. Overnight cultures were back-diluted to an initial density of OD_600_ 0.01 in fresh growth medium (LB + 25 µg/mL apramycin + 50 ng/mL aTc) and grown with shaking at 37°C for 3.5 h. Following outgrowth, RNA was collected from 1 mL of cells using Zymo Direct-zol RNA Miniprep kit (Zymo, cat. no. R2062) according to the manufacturer’s recommendations, including on-column DNase digestion. Purified RNA was converted to cDNA using the LunaScript RT Master Mix Kit (NEB, cat. no. E3025) in a 20 µL reaction containing 1 x LunaScript RT Master Mix, 1 µL of random hexamers (250 ng/µL), and 1 µg total RNA. Reactions were incubated according to the manufacturer’s recommendations for random primers (25°C for 2 min, 55°C for 10 min, 95°C for 1 min). The cDNA was diluted 10-fold with nuclease-free water, and 2 µL of the diluted cDNA was used for each qPCR reaction. qPCR reactions were performed in technical triplicate using iTaq Universal SYBR Green SuperMix (Bio-Rad, cat. no. 1725122) on an ABI QuantStudio 3 instrument (Applied Biosystems). For each target gene, primer efficiencies were determined via serial dilutions and melting curve analyses. Data analysis was supported by the QuantStudio software. Transcript abundance for each target was measured relative to the abundance of the *recA* transcript. Data are depicted as the relative transcript abundance compared to wild-type cells harboring the empty vector. The data are presented as the mean of three biological triplicate cultures and were calculated using the comparative threshold cycle (*C_T_*) method (2^^-∆∆CT^) as described previously (19). qRT-PCR experiments were conducted with biological triplicate cultures and independently repeated twice. Data from a single representative experiment run are shown. Error bars represent one standard deviation of the mean. Results were analyzed for significance using two-tailed *t*-tests.

### H33342 accumulation assay

H33342 accumulation experiments were conducted as described previously (52). Overnight cultures (grown in LB) were refreshed in fresh LB medium containing 25 µg/mL apramycin for plasmid maintenance and 50 ng/mL aTc to induce expression of HemZ, as needed. The cultures were adjusted to a starting density of 0.01 OD_600_ units and grown to exponential phase (3 h 15 min). At this point, cells from 5 mL of culture were pelleted, washed once with 1 mL sterile PBS, pelleted again, and resuspended to a density of 0.5 OD_600_ units in PBS. Aliquots of the prepared cells (180 µL) were then added in technical quadruplicate wells to a black-walled, clear bottom 96-well plate containing H33342 dye at a final concentration of 2.5 µM, in a total volume of 200 µL. The accumulation of H33342, which is fluorescent when bound to DNA, was monitored at ambient temperature in a Tecan M200 microplate reader with excitation/emission wavelengths of 355 nm and 460 nm, respectively. Readings were taken every 3 min for 3 h. The technical replicates for each strain/plasmid condition at each were averaged and are plotted over time (error bars are omitted for clarity). As controls, the prepared cells were heated at 100°C for 10 min prior to addition of H33342 dye (S8 Fig., “heat-killed cells”), and the reactions also included carbonyl cyanide-m-chlorophenyl hydrazone (CCCP) at a final concentration of 5 µM (S8 Fig., “cells + CCCP”). H33342 accumulation assays were repeated independently three times with data shown from a single representative experiment.

### Irgasan growth curves

Irgasan growth curves were conducted in a 96-well dish with triplicate wells for each strain/plasmid condition tested. Overnight cultures, grown in M9-succinate medium with apramycin (25 µg/mL), were diluted (1:200) into a final volume of 200 µL M9-succinate + apramycin with or without 250 nM irgasan. The plates were incubated at 37°C in a Spectra Max 190 plate reader. Growth was monitored at 37°C for 24 h with OD_600_ readings captured every 15 min. The plate was shaken for 5 s prior to each read. Data are plotted as the mean cell density value for the triplicate wells. The experiment was repeated independently three times (i.e., different days with newly transformed plasmids). The results of a single representative run are shown.

## Data Availability

The RIL-seq sequencing data, including the raw sequencing reads and processed files in bigwig format, are available at NCBI under GEO accession number GSE295534. The raw sequencing reads and processed files in bigwig format for total RNA-seq samples are available NCBI under GEO accession number GSE295535. The processed RIL-seq and RNA-seq data are available via an IGV web app at https://tinyurl.com/4xa9y3y2 (exponential phase) and https://tinyurl.com/4ae3d4y4 (stationary).

## SUPPLEMENTAL MATERIAL

Data set S1 mbio.03231-25-s0001.xlsx RIL-seq data for exponential phase and stationary phase.

Supplemental material mbio.03231-25-s0002.pdf Figures S1 to S8, Tables S1 to S6, and supplemental methods.

ASM does not own the copyrights to Supplemental Material that may be linked to, or accessed through, an article. The authors have granted ASM a non-exclusive, world-wide license to publish the Supplemental Material files. Please contact the corresponding author directly for reuse.
