# Supplementary material for: Hfq orchestrates a robust RNA-RNA interaction network in Acinetobacter baumannii
Source: mBio. 2025 Dec 17;17(1):e03231-25. doi: 10.1128/mbio.03231-25 (PMC12802169; doi:10.1128/mbio.03231-25)
Supplement: Supplemental material — Figures S1 to S8, Tables S1 to S6, and supplemental methods. [file mbio.03231-25-s0002.pdf]

Supplementary Materials

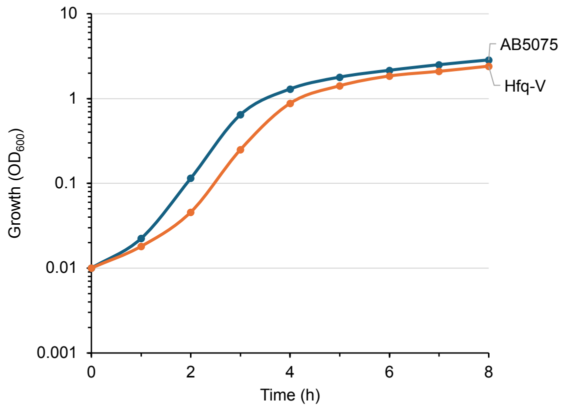

Supplementary Figure S1

Figure S1. Growth curve of wild-type AB5075 and AB5075 *hfq-V.* Triplicate overnight cultures of WT AB5075 and its isogenic *hfq-V* derivative (Hfq-V) were back-diluted in LB to an OD_{600} of 0.01 and grown at 37˚C with shaking. Growth was monitored by recording the OD_{600} at the indicated time points. Data are plotted as the mean of the three biological replicate cultures.

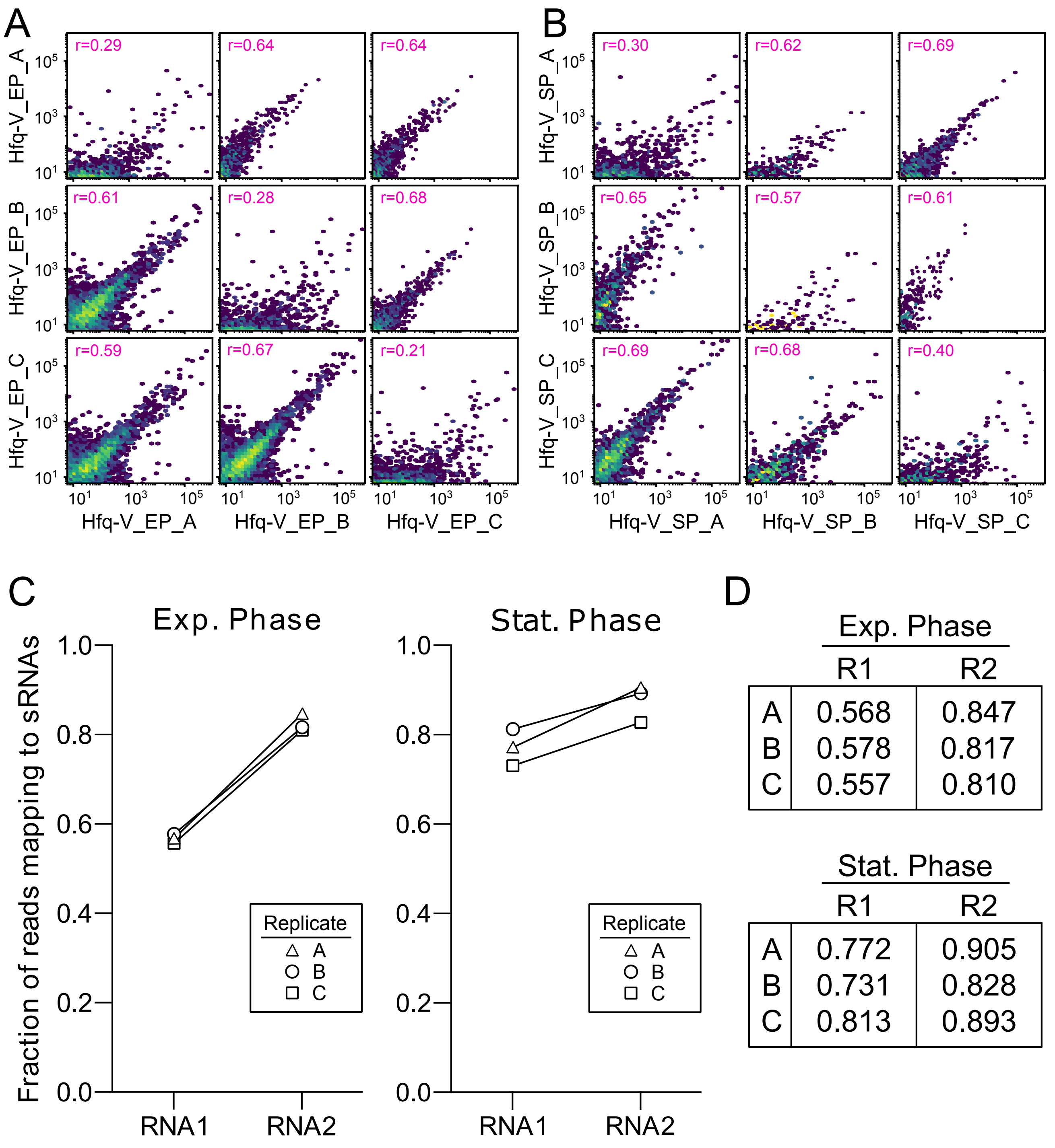

Exp. Phase

	R1	R2
A	0.568	0.847
B	0.578	0.817
C	0.557	0.810

Stat. Phase

	R1	R2
A	0.772	0.905
B	0.731	0.828
C	0.813	0.893

Supplementary Figure S2. Assessment of RIL-seq dataset reproducibility. Reproducibility of the RIL-seq datasets between replicates for **(A)** exponential phase and **(B)** stationary phase was evaluated essentially as described previously (1, 2). For each sample, sequenced fragments were binned into 100-bp fragments across the genome with each point in the plot representing the number of fragments mapping to each genomic bin. Color scales with intensity and ranges from blue (low number of fragments) to yellow (high number of fragments). The plots positioned above the diagonal (i.e., the upper right three plots for each matrix) represent reproducibility amongst the significant chimeric

fragments in each dataset; plots positioned along the diagonal represent intra-library comparisons between chimeric fragments and single fragments; and plots below the diagonal (lower left three plots) represent reproducibility between the single fragments. The Spearman correlation coefficients, r, are reported for each scatter plot. **(C)** Fraction of reads mapping to sRNAs in the RNA1 position and the RNA2 position in exponential phase (left) and stationary phase (right) for the sequencing library from each of the three biological replicates. **(D)** Data from (C) presented in tabular form. R1, fraction of reads mapping to sRNAs in the RNA 1 read position; R2, fraction of reads mapping to sRNAs in the RNA 2 read position. No statistical differences were detected between the fraction of reads mapping as the RNA 1 vs RNA 2 position for either the exponential or stationary phase by Wilcoxon Signed Rank test.

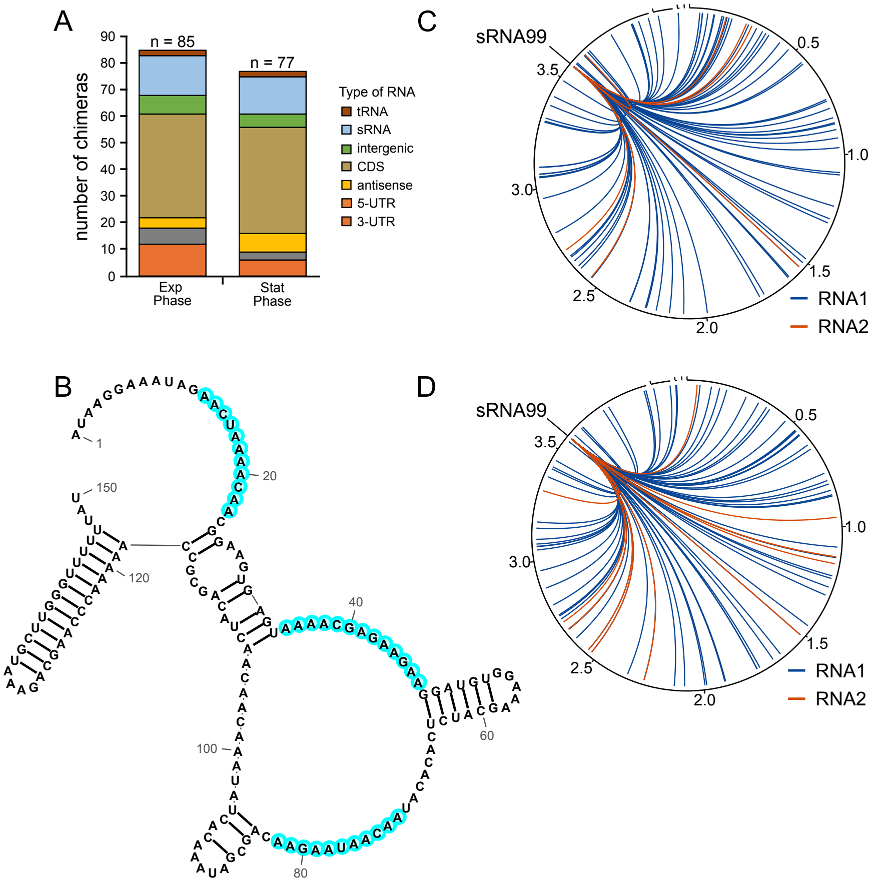

Supplementary Figure S3. sRNA99 is a dominant RNA in the RILseq datasets. **(A)** Types of RNA interaction partners for sRNA99. Graphs depict the distribution of annotation types found in chimeric pairings with sRNA99 in the RNA1 condition for the exponential phase (Exp Phase) and stationary phase (Stat Phase) RIL-seq datasets. **(B)** Predicted secondary structure of sRNA99. The primary sequence of sRNA99 was analyzed via RNAfold (3) and the resulting structure prediction visualized using RNA Canvas web app (4). Nucleotides highlighted in blue indicate potential A-rich motifs. **(C)** and **(D)** Circos (5) plots depicting sRNA99-containing chimeras for exponential phase (C) and stationary phase (D). Chimeras where sRNA99 was in the RNA1 position are drawn as blue links and those where sRNA99 was in the RNA2 position are drawn in orange.

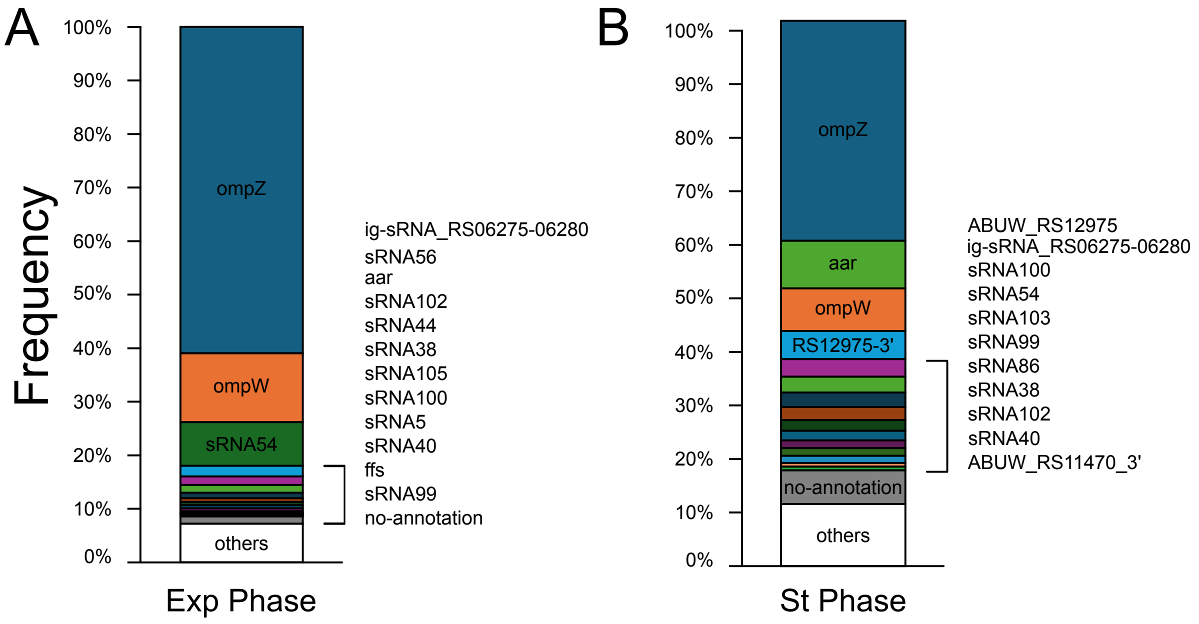

Supplementary Figure S4. Relative abundance of RNA species on Hfq. RNAs interacting with Hfq as single fragments (i.e., non-chimeric RNAs) were mapped to their corresponding genomic location. The graph in **(A)** depicts the top 10 most abundant RNA species detected in the exponential phase dataset. The graph in **(B)** depicts the top 10 most abundant RNA species detected in the stationary phase dataset.

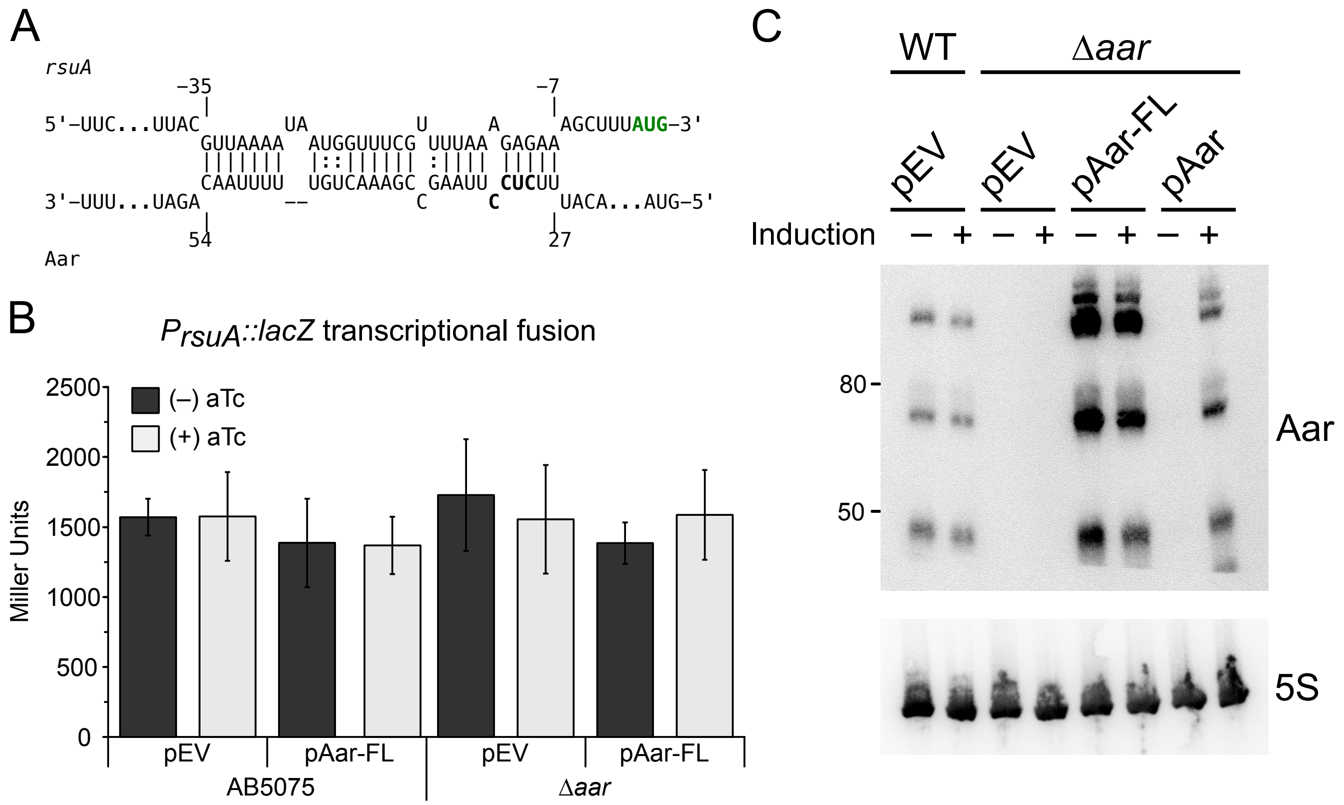

Supplementary Figure S5. The *rsuA* transcript is an Aar target. **(A)** Aar-RsuA interaction predicted by IntaRNA (6). The *rsuA* start codon is in green bold-face font. The previously identified Aar seed sequence is in bold text (7). Numbering for *rsuA* is depicted in relationship to the first nucleotide of the *rsuA* start codon. **(B)** Aar does not significantly alter *rsuA* promoter activity. Beta-galactosidase assays were conducted as described in the main text. Briefly, the indicated reporter strains harboring the indicated plasmids were grown in LB + apramycin, in the presence or absence of 50 ng/mL anhydrous tetracycline (aTc) for 6 hours and assayed for beta-galactosidase activity. The experiment was conducted with biological triplicate cultures and repeated independently at least two times. Results shown are from a representative experiment run. **(C)** Northern blot analysis of Aar in WT, Aar deletion strain (Δ*aar*), and complementing plasmids. Cultures grown to early stationary phase (OD_{600} ≈ 1.8-2.2) were exposed to vehicle control (DMSO, (–) aTc) or inducer (50 ng/mL aTc, (+) aTc) for 20 minutes, after which time total RNA was collected. The RNA was resolved through a denaturing acrylamide gel, transferred to a positively charged nylon membrane and probed with oligonucleotides complementary to the indicated RNA species. The Aar probe was labelled with ^{32}P and imaged via autoradiography. The 5S rRNA probe was labelled with IRDye-700 and imaged via near-IR. The migration of single strand RNA standards are indicated in bp to the left of the blots. pEV, empty vector (pMJG598); pAar-FL, pMJG598 with Aar expression driven by the native Aar promoter; pAar, pMJG598 with Aar expression driven by the tetracycline responsive promoter in pMJG598.

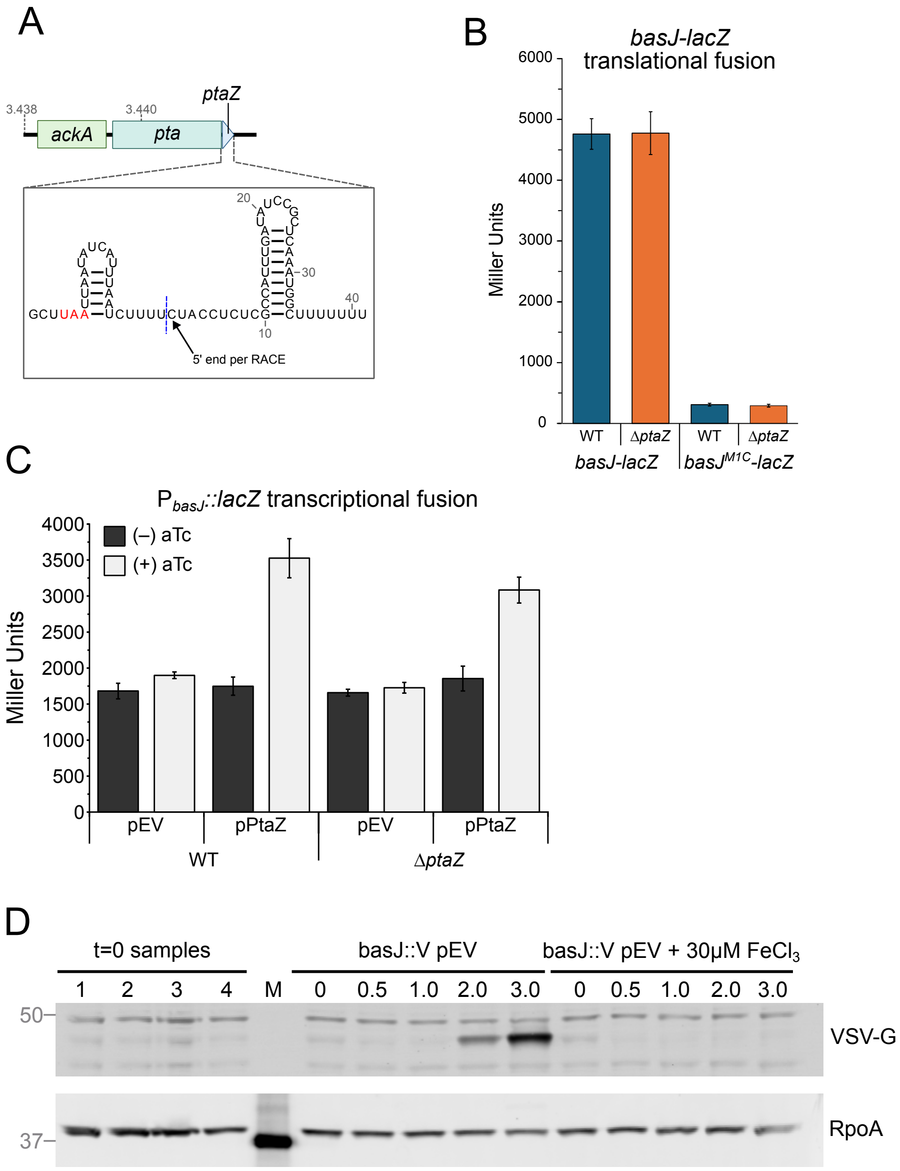

Supplementary Figure S6. PtaZ is derived from the 3′-UTR of the *ackA-pta* transcript. **(A)** Schematic of the *ackA-pta-ptaZ* chromosomal locus. Inset below shows the predicted secondary structure of the *ptaZ* region, the stop codon for the *pta* open reading frame is depicted in red font. Blue line indicates the starting point of PtaZ as determined by 5′-RACE. Numbers above schematic indicate chromosome position (in megabases). **(B)** β-galactosidase activity (in Miller Units) of AB5075 and AB5075 Δ*ptaZ* cells harboring translational fusion between the native *basJ* promoter region (left data column) and the M1C mutant derivative of the *basJ* promoter region. For the β-galactosidase assays, cells were grown for 6 hours in M9-Succinate minimal medium and were independently repeated at least twice. Data from a single representative experiment are shown as the mean activity for biological triplicate cultures with error bars representing one standard deviation of the mean. No statistical differences were noted between reporter activities between WT and the Δ*ptaZ* mutant for either reporter by two-tailed t-tests. **(C)** Beta-galactosidase assays were conducted as described in the main text. Briefly, the indicated reporter strains harboring the indicated plasmids were grown in M9-succinate medium + apramycin, in

the presence or absence of 5 ng/mL aTc for 6 hours and assayed for beta-galactosidase activity. The experiment was conducted with biological triplicate cultures and repeated independently at least two times. Results shown are from a representative experiment run. **(D)** Western blot analysis following switch from iron-replete (LB) to iron-limited (M9-Succinate) media. Cultures of WT BasJ-V or Δ*ptaZ* BasJ-V containing the indicated plasmids were grown for 3 hours in LB, washed in M9-salts and resuspended in M9-Succinate medium containing 5 ng/mL aTc and grown with shaking at 37˚C. The left four lanes contain the t = 0 samples (i.e., immediately following resuspension in M9+succinate). Lane 1, WT *basJ-V* pEV; Lane 2, Δ*ptaZ basJ-V* pEV; Lane 3, Δ*ptaZ basJ-V* pPtaZ; Lane 4, WT *basJ-V* pEV + 30 μM $FeCl_3$. The 5th lane (M) contain the molecular weight marker. Lanes to the right of the Marker lane were collected from WT *basJ-V* pEV grown without (middle lanes) or with (right-most lanes) the addition of 30 μM $FeCl_3$. Cells were collected at the indicated time points (in hours) and analyzed by Western Blot as described in the main text.

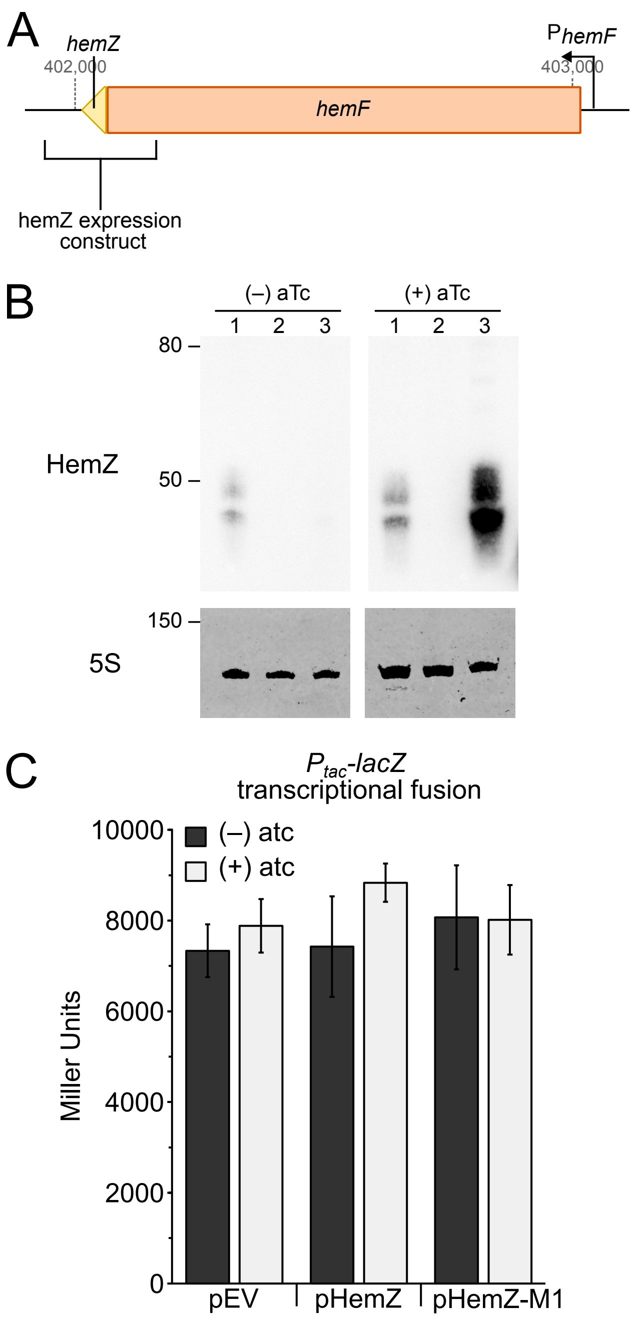

Supplementary Figure S7. HemZ is derived from the 3′-UTR of the *hemF* transcript. **(A)** Schematic of the *hemF-hemZ* locus. Numbers in grey indicate chromosomal position (in bp). The boundaries for the expression construct used to ectopically express HemZ (pHemZ) are indicated. **(B)** Northern blot analysis for the pHemZ expression plasmid. Exponentially growing cultures (OD_{600} ≈ 0.4-0.6) were exposed to vehicle control (DMSO, (–) aTc) or inducer (50 ng/mL aTc, (+) aTc) for 20 minutes, after which time total RNA was collected. The RNA was resolved through a denaturing acrylamide gel, transferred to a positively charged nylon membrane and probed with oligonucleotides complementary to the indicated RNA species. The HemZ probe was labelled with ^{32}P and imaged via autoradiography. The 5S rRNA probe was labelled with IRDye-700 and imaged via near-IR. The migration of single strand RNA standards are indicated in bp to the left of the blots. Lane 1: wild type AB5075 with an

empty vector (pEV); lane 2: Δ*hemZ* pEV; lane 3: Δ*hemZ* pHemZ. **(C)** HemZ does not alter activity of the *Ptac-lacZ* transcriptional reporter. Beta-galactosidase assays were conducted as described in the main text. Briefly, the indicated reporter strains harboring the indicated plasmids were grown in LB + apramycin with 0.5 mM IPTG, in the presence or absence of 50 ng/mL aTc for 4 hours and assayed for beta-galactosidase activity. The experiment was conducted with biological triplicate cultures and repeated independently at least two times. Results shown are from a representative experiment run.

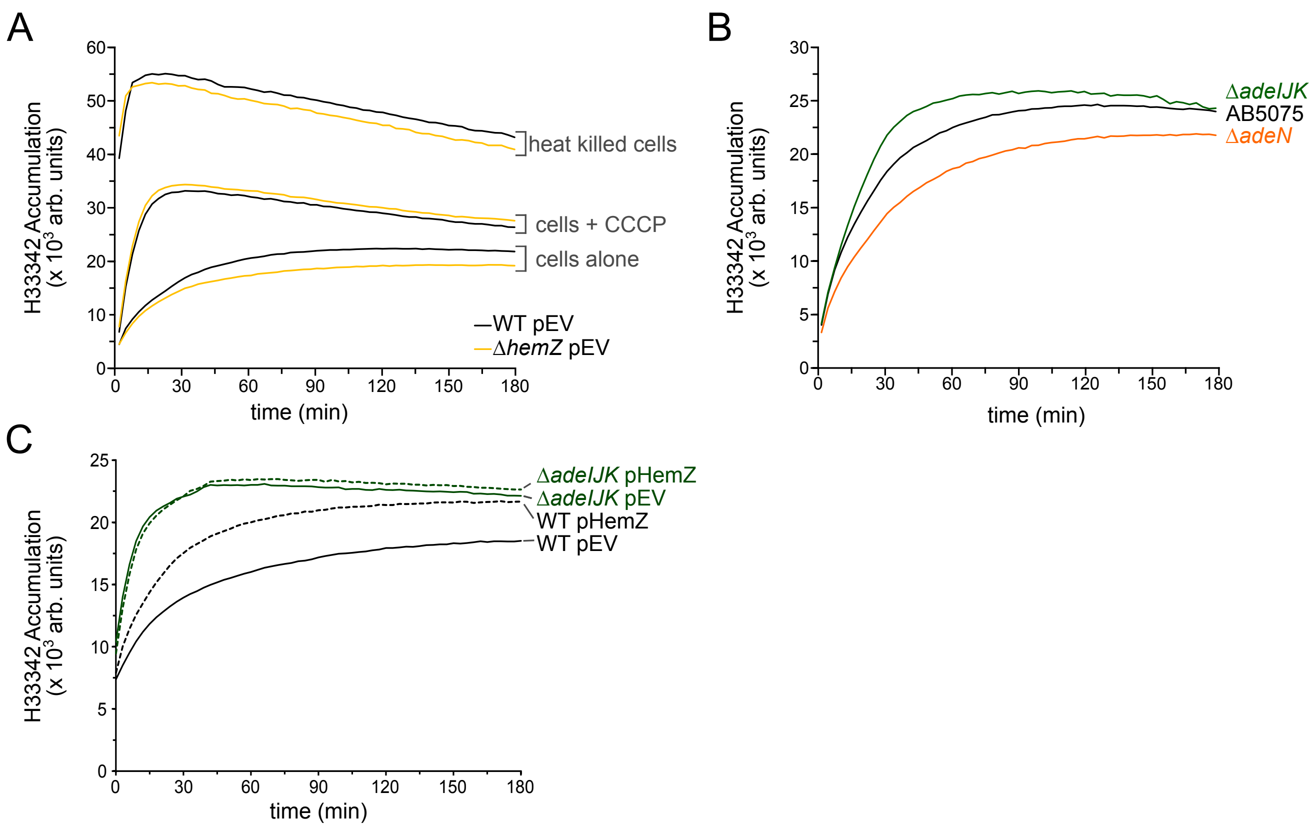

Supplementary Figure S8: H33342 Accumulation Assay. **(A)** Exponentially growing cells (OD_{600} ≈ 0.6) of WT AB5075 with empty vector (pEV; black lines) or Δ*hemZ* with empty vector (Δ*hemZ* pEV, black line) were washed in PBS and either directly mixed with H33342 dye at a final concentration of 25 µM (cells alone), mixed with both 5 µM CCCP and 25 µM H33342 dye (cells + CCCP), or heated to 100˚C for 10 minutes prior to the addition of 25 µM H33342 dye (heat killed cells). In **(B)** and **(C),** the indicated control strains were directly mixed with H33342 dye (25 µM final concentration). The reactions were carried out in a 96-well dish in technical quadruplicate. The assay plates were monitored for H33342 fluorescence (excitation: 355 nm, emission: 460 nm) in a microplate reader with readings every three minutes for a total of three hours at ambient temperature. Data is displayed as the mean fluorescence value for each strain/plasmid condition. Cultures in A and C included 50 ng/mL aTc to induce expression from P_{tetA} on the vector. The experiment was repeated independently three times with a single, representative experiment run displayed.

Supplementary Tables

Supplementary Table S1. Essential, Virulence, and Antibiotic Resistance Genes identified by RIL-seq

Chimera	RNA 1 Locus Tag	RNA 1 Gene Name	RNA 2 Name	# of interactions
**Exponential Phase**				
Essential Genes				
27	*ABUW_RS07855*	*miaA*	ABUW_RS10475-3'	599
34	*ABUW_RS07450*		sRNA100	434
45	*ABUW_RS11025*		ABUW_RS06280-3'	241
59	*ABUW_RS04260*	*sdhC*	OmpZ	179
67	*ABUW_RS14815*		ABUW_RS14740-3'	161
92	*ABUW_RS00805*	*guaB*	sRNA100	109
93	*ABUW_RS18890*	*dnaK*	ABUW_RS06280-3'	108
121	*ABUW_RS03070*	*folC*	sRNA64	83
130	*ABUW_RS04225*		ABUW_RS01825-3'	75
136	*ABUW_RS02090*	*rplO*	ABUW_RS15185_3'	71
173	*ABUW_RS10900*	*rpsA*	as-sRNA_ABUW_RS20565	55
192	*ABUW_RS08470*	*ispC*	sRNA100	47
204	*ABUW_RS04180*	*serS*	Art53	43
218	*ABUW_RS00730*	*murC*	Aar	39
221	*ABUW_RS05500*	*mnmA*	OmpZ	39
253	*ABUW_RS08240*		as-sRNA_ABUW_RS17700	34
288	*ABUW_RS00810*	*glmM*	as-sRNA_ABUW_RS17700	28
296	*ABUW_RS08480*	*bamA*	ABUW_RS14740-3'	27
298	*ABUW_RS10970*		Aar	27
306	*ABUW_RS07590*	*ppsA*	ABUW_RS14740-3'	26
308	*ABUW_RS04290*	*lpdA*	sRNA64	26
317	*ABUW_RS08885*	*kdsA*	as-sRNA_ABUW_RS20565	25
337	*ABUW_RS18190*	*atpE*	sRNA101	23
354	*ABUW_RS15375*	*nuoM*	ABUW_RS14740-3'	22
368	*ABUW_RS15090*	*fabG*	Art53	21
374	*ABUW_RS17035*		ABUW_RS01510-3'	21
392	*ABUW_RS02105*	*rpsM*	as-ABUW_RS03670	19
393	*ABUW_RS02100*	*rpmJ*	ABUW_RS01510-3'	19
399	*ABUW_RS15980*	*thrS*	ABUW_RS03025-3'	19
415	*ABUW_RS07575*	*cyoB*	sRNA99	18
416	*ABUW_RS16525*	*clpX*	sRNA54	18
419	*ABUW_RS01455*	*aroB*	ABUW_RS06280-3'	18
425	*ABUW_RS08795*	*rpiA*	ompA	17
445	*ABUW_RS14720*	*fur*	rpsE	17
485	*ABUW_RS04975*	*dapD*	ABUW_RS14740-3'	16
493	*ABUW_RS17205*		tRNA-Met	16
495	*ABUW_RS00845*		sRNA103	16
505	*ABUW_RS02425*	*rplM*	as-sRNA_ABUW_RS17700	15
522	*ABUW_RS03065*	*accD*	ABUW_RS18620	15
558	*ABUW_RS03095*	*secA*	ABUW_RS01825-3'	14
562	*ABUW_RS04285*	*odhB*	sRNA100	14
564	*ABUW_RS18745*		ABUW_RS14740-3'	14
573	*ABUW_RS11165*	*tkt*	rpsJ	14
602	*ABUW_RS16675*	*rpmB*	wza	13
607	*ABUW_RS15040*	*rplY*	tRNA-Met	13
608	*ABUW_RS17195*		ABUW_RS16520	13
613	*ABUW_RS02420*	*rpsI*	tRNA-Met	13
622	*ABUW_RS05400*		3-ETS_tRNA-Phe	13
626	*ABUW_RS01260*	*ompR*	putA	13

627	*ABUW_RS08270*	*aroQ*	ABUW_RS07925-3'	13
638	*ABUW_RS17215*		ABUW_RS01825-3'	13
646	*ABUW_RS17965*	*parC*	ABUW_RS17960	13
Virulence Genes				
32	*ABUW_RS07050*		ABUW_RS01510-3'	470
33	*ABUW_RS00155*	*ppc*	ABUW_RS14740-3'	452
114	*ABUW_RS18620*		sRNA100	87
128	*ABUW_RS16670*		sRNA105	76
132	*ABUW_RS07865*	*eamA*	sRNA100	74
138	*ABUW_RS17705*		ABUW_RS01510-3'	71
176	*ABUW_RS18595*		pilA	54
216	*ABUW_RS03195*	*omp38*	ABUW_RS09875	39
241	*ABUW_RS03565*		ABUW_RS01825-3'	35
282	*ABUW_RS08405*		ABUW_RS01510-3'	29
289	*ABUW_RS09560*		ABUW_RS14740-3'	28
297	*ABUW_RS00670*		ABUW_RS14740-3'	27
314	*ABUW_RS13305*	*crp*	3-utr-ABUW_RS12390	25
338	*ABUW_RS03005*		ABUW_RS14900	23
347	*ABUW_RS18610*		ABUW_RS15185_3'	22
352	*ABUW_RS11000*		sRNA40	22
371	*ABUW_RS04135*	*adeI*	HemZ	21
381	*ABUW_RS13350*	*csrA*	CsrB	20
394	*ABUW_RS16730*		ABUW_RS06280-3'	19
400	*ABUW_RS15000*		sRNA100	19
408	*ABUW_RS10735*		ABUW_RS08690	18
420	*ABUW_RS10925*	*ung*	Aar	18
435	*ABUW_RS16475*		sRNA44	17
443	*ABUW_RS05515*	*dacC*	ABUW_RS14740	17
479	*ABUW_RS04325*		ABUW_RS14740-3'	16
524	*ABUW_RS09270*		ABUW_RS06280-3'	15
571	*ABUW_RS15285*	*metG*	ABUW_RS14740-3'	14
575	*ABUW_RS15825*		ig-ABUW_RS06160-ABUW_RS06165	14
584	*ABUW_RS12255*		3-utr_ABUW_RS03670	14
604	*ABUW_RS13305*	*crp*	argA	13
606	*ABUW_RS18605*		ig-sRNA_ABUW_RS14270-RS14275	13
617	*ABUW_RS13705*		5-utr_oxa23	13
639	*ABUW_RS16420*	*pilW*	sRNA103	13
655	*ABUW_RS16765*		OmpZ	13
Antibiotic Resistance Genes				
26	*ABUW_RS02765*	*oxa23*	PtaZ	636
132	*ABUW_RS07865*	*eamA*	sRNA100	74
178	*ABUW_RS19270*	*dfrA7*	3-utr_ABUW_RS06415	53
242	*ABUW_RS00170*	*arpB*	ABUW_RS14740-3'	35
278	*ABUW_RS04920*		CsrB	29
371	*ABUW_RS04135*	*adeI*	HemZ	21
421	*ABUW_RS19295*	*aph6*	tRNA-Met	18
483	*ABUW_RS00970*		sRNA64	16
508	*ABUW_RS19280*	*ges11*	3-utr_ABUW_RS03670	15
514	*ABUW_RS05550*		ig-sRNA_ABUW_RS14270-RS14275	15
519	*ABUW_RS19315*	*aadB*	ABUW_RS01510-3'	15
574	*ABUW_RS11280*		3-utr_ABUW_RS00295	14
**Stationary Phase**				
Essential Genes				
63	*ABUW_RS04225*		ABUW_RS01825-3'	88
133	*ABUW_RS00750*	*ftsZ*	sRNA103	40

159	*ABUW_RS07450*		CsrB	34
167	*ABUW_RS18745*		sRNA54	32
204	*ABUW_RS18890*	*dnaK*	ABUW_RS06280-3'	24
210	*ABUW_RS11025*		ABUW_RS06280-3'	23
219	*ABUW_RS15410*	*nuoF*	Aar	22
234	*ABUW_RS17505*	*secE*	OmpZ	21
238	*ABUW_RS03095*	*secA*	Aar	20
271	*ABUW_RS07855*	*miaA*	ABUW_RS03025-3'	18
273	*ABUW_RS04290*	*lpdA*	sRNA64	18
299	*ABUW_RS15930*	*pheT*	sRNA103	16
315	*ABUW_RS15185*		smc	15
323	*ABUW_RS00810*	*glmM*	as-sRNA_ABUW_RS17700	15
342	*ABUW_RS15430*	*ndhC*	sRNA103	14
370	*ABUW_RS10900*	*rpsA*	as-sRNA_ABUW_RS20565	14
381	*ABUW_RS04230*	*rpoD*	Aar	13
443	*ABUW_RS03075*		ABUW_RS14045	12
480	*ABUW_RS02425*	*rplM*	Art20	11
485	*ABUW_RS16610*	*lgt*	ABUW_RS07925-3'	11
490	*ABUW_RS17785*	*ribD*	OmpZ	11
501	*ABUW_RS02125*	*rplQ*	as-ABUW_RS09980	11
540	*ABUW_RS01380*	*rsmH*	ABUW_RS19280	11
Virulence Genes				
56	*ABUW_RS13005*		sRNA99	115
60	*ABUW_RS08040*	*mcsS*	katE	108
61	*ABUW_RS10010*		SabS	97
74	*ABUW_RS09270*		ABUW_RS06280-3'	76
88	*ABUW_RS11870*		ig-sRNA_ABUW_RS07165-RS07170	61
124	*ABUW_RS03195*	*ompA*	SabS	44
127	*ABUW_RS16670*		sRNA105	42
131	*ABUW_RS10735*		ABUW_RS08690	40
160	*ABUW_RS07865*	*eamA*	sRNA 100	33
230	*ABUW_RS13010*		sRNA103	21
259	*ABUW_RS18620*		sRNA100	18
338	*ABUW_RS07145*		PtaZ	14
361	*ABUW_RS13805*		3-utr_ABUW_RS10585	14
388	*ABUW_RS13590*		3-utr_ABUW_RS06415	13
405	*ABUW_RS09075*		int-sRNA_ABUW_RS08920	13
421	*ABUW_RS17385*	*fadB*	ig-sRNA_ABUW_RS06275-06280	12
429	*ABUW_RS00370*	*hutC*	ABUW_RS15185-3'	12
439	*ABUW_RS07790*		Aar	12
452	*ABUW_RS08380*		ABUW_RS19125	12
495	*ABUW_RS12325*	*paaA*	ABUW_RS06280-3'	11
496	*ABUW_RS12255*		sRNA103	11
541	*ABUW_RS01735*	*astB*	3-ETS_tRNA-Phe	11
557	*ABUW_RS15160*	*otsB*	sRNA64	11
Antibiotic Resistance Genes				
41	*ABUW_RS02765*	*oxa23*	PtaZ	205
55	*ABUW_RS19315*	*aadB*	sRNA103	116
101	*ABUW_RS00170*		sRNA99	51
160	*ABUW_RS07865*	*eamA*	sRNA100	33
281	*ABUW_RS00485*	*aciT*	ABUW_RS10025	17
308	*ABUW_RS14265*		ABUW_RS06280-3'	15
329	*ABUW_RS09380*		CsrB	15
428	*ABUW_RS19280*	*ges11*	ig-sRNA_groL-dgkA	12
523	*ABUW_RS04920*		CsrB	11

Supplementary Table S2. Novel candidate sRNAs identified by RIL-seq

sRNA name	Locus tag	Strand	Start	Stop
ABUW_RS00475_3'	3-sRNA_ABUW_RS00475	-	107564	107609
ig-sRNA_ABUW_RS00840-RS00845	ig-sRNA_ABUW_RS00840-RS00845	+	189267	189317
ABUW_RS01510_3'	3-sRNA_ABUW_RS01510	-	330834	330924
ABUW_RS01825_3'	3-sRNA_ABUW_RS01825	-	395422	395480
HemZ	3-sRNA_ABUW_RS01850	-	402049	402099
ig-sRNA_ABUW_RS02145-RS02150	ig-sRNA_ABUW_RS02145-RS02150	+	449981	450061
ig-sRNA_ABUW_RS2735-02740	ig-sRNA_ABUW_RS02735-RS02740	+	557470	557514
ABUW_RS03025_3'	3-sRNA_ABUW_RS03025	-	615529	615606
ig-sRNA_ABUW_RS19685-RS03310	ig-sRNA_ABUW_RS19685-RS03310	+	675795	675862
as-sRNA_ABUW_RS20565	as-sRNA_ABUW_RS20565	-	776726	776791
as-sRNA_ABUW_RS03925	as-sRNA_ABUW_RS03925	-	790854	790947
ig-sRNA_groL-dgkA	ig-sRNA_ABUW_RS04505-RS04510	+	925276	925346
ig-sRNA_ABUW_RS06275-06280	ig-sRNA_ABUW_RS06275-RS06280	+	1307227	1307321
ABUW_RS06280_3'	3-sRNA_ABUW_RS06280	+	1308094	1308141
as-sRNA_ABUW_RS06870	as-sRNA_ABUW_RS06870	-	1408236	1408301
ig-sRNA_ABUW_RS07165-RS07170	ig-sRNA_ABUW_RS07165-RS07170	+	1460023	1460078
ABUW_RS07925_3'	3-sRNA_ABUW_RS07925	-	1618921	1619001
ABUW_RS08635_3'	3-sRNA_ABUW_RS08635	-	1772916	1772962
int-sRNA_ABUW_RS08920	int-sRNA_ABUW_RS08920	+	1829166	1829221
int-sRNA_ABUW_RS09115	int-sRNA_ABUW_RS09115	+	1872420	1872475
ABUW_RS10010_3'	3-sRNA_ABUW_RS10010	-	2042667	2042773
ABUW_RS10475_3'	3-sRNA_ABUW_RS10475	-	2138790	2138883
ABUW_RS10850_3'	3-sRNA_ABUW_RS10850	+	2221412	2221485
ABUW_RS11470_3'	3-sRNA_ABUW_RS11470	+	2342075	2342144
ig-sRNA_ABUW_RS12420-12425	ig-sRNA_ABUW_RS12420-RS12425	-	2547545	2547586
ABUW_RS12725_3'	3-sRNA_ABUW_RS12725	-	2618467	2618548
ABUW_RS12975_3'	3-sRNA_ABUW_RS12975	-	2666247	2666325
ig-sRNA_ABUW_RS14270-RS14275	ig-sRNA_ABUW_RS14270-RS14275	-	2955477	2955527
int-sRNA_ABUW_RS14485	int-sRNA_ABUW_RS14485	-	2999962	3000016
ABUW_RS14740_3'	3-sRNA_ABUW_RS14740	-	3063582	3063714
ABUW_RS15185_3'	3-sRNA_ABUW_RS15185	-	3149041	3149114
PtaZ	3-sRNA_ABUW_RS16505	+	3441647	3441688
ABUW_RS16550_3'	3-sRNA_ABUW_RS16550	+	3454102	3454151
OmpZ	3-sRNA_ABUW_RS17440	-	3641853	3641936
as-sRNA_ABUW_RS17700	as-sRNA_ABUW_RS17700	-	3693935	3693980
ig-sRNA_ABUW_RS19585-19030[a]	ig-sRNA_ABUW_RS19585-RS19030	-	83404	83508
ABUW_RS19600_3'[b]	3-sRNA_ABUW_RS19600	-	1751	1870
ig-sRNA_ABUW_RS19600-19605[b]	ig-sRNA_ABUW_RS19600-RS19605	+	2561	2651

Notes: a) located on plasmid p1AB5075
b) located on plasmid p2AB5075

Supplementary Table S3. Bacterial Strains used in this study

**Strain**	**Source or Reference**
Escherichia coli DH5α F'-lacI^q	Lab Stock
E. coli LW264	(8)
E. coli SM10 λpir$^+$ pTNS3	Lab Stock
E. coli Top10 pRK2013	Lab Stock
Acinetobacter baumannii AB5075-UW	(9)
A. baumannii ATCC 17978	Lab Stock
A. baumannii AYE	(10)
A. baumannii ACICU	(11)
A. baumannii ACI-1; MDR clinical isolate	(12)
A. baumannii ACI-14; MDR clinical isolate	Lance R. Peterson
AB5075 *hfq-VSVG*	This study
AB5075 *attTn7-dCas9*	(13)
AYE *attTn7-dCas9*	This study
ACICU *attTn7-dCas9*	This study
ACI-1 *attTn7-dCas9*	This study
ACI-14 *attTn7-dCas9*	This study
ATCC 17978 *attTn7-dCas9*	This study
AB5075 *hfq-V attTn7-dCas9*	This study
AB5075 *carO-VSVG*	This study
AB5075 Δ*aar*	This study
AB5075 Δ*aar carO-VSVG*	This study
AB5075 Δ*aar carO-M1C-VSVG*	This study
ATCC 17978 *carO-VSVG*	This study
AB5075 *attTn7-rsuA::lacZ* translational fusion	This study
AB5075 Δ*aar attTn7-rsuA::lacZ* translational fusion	This study
AB0575 Δ*ptaZ*	This study
AB5075 *basJ-VSVG*	This study
AB5075 Δ*ptaZ basJ-VSVG*	This study
AB5075 *attTn7-basJ::lacZ* translational fusion	This study
AB5075 Δ*ptaZ attTn7-basJ::lacZ* translational fusion	This study
AB5075 *attTn7-basJ-M1C::lacZ* translational fusion	This study
AB5075 Δ*ptaZ attTn7-basJ-M1C::lacZ* translational fusion	This study
AB5075 Δ*hemZ*	This study
AB5075 *attTn7-Ptac-adeI::lacZ* translational fusion	This study
AB5075 Δ*hemZ attTn7-Ptac-adeI::lacZ* translational fusion	This study
AB5075 *attTn7-Ptac-adeI-M1C::lacZ* translational fusion	This study
AB5075 Δ*hemZ attTn7-Ptac-adeI-M1C::lacZ* translational fusion	This study
AB5075 *attTn7-Ptac::lacZ* transcriptional fusion	(13)
AB5075 Δ*hemZ attTn7-Ptac::lacZ* transcriptional fusion	This study
AB5075 Δ*adeIJK*	This study
AB5075 Δ*adeN*	This study
AB5075 *attTn7-rsuA::lacZ* transcriptional fusion	This study
AB5075 Δ*aar attTn7-rsuA::lacZ* transcriptional fusion	This study
AB5075 *attTn7-basJ::lacZ* transcriptional fusion	This study
AB5075 Δ*ptaZ attTn7-basJ::lacZ* transcriptional fusion	This study

Supplementary Table S4. Plasmids used in this study

**Plasmid**	**Description**	**Source**
Plasmids to introduce mutations to A. baumannii chromosome		
pMJG42	Allele exchange vector	(8)
pMJG42.*hfq-VSVG*	Construction of VSV-G tag at native *hfq* locus	This study
pMJG42.*carO-VSVG*	Construction of VSV-G tag at native *carO* locus	This study
pMJG42.Δ*aar*	Replacement of *aar* sequence with tR′ terminator	This study
pMJG42.*carO*^{M1C}*-VSVG*	Introduction of M1C allele of *carO-VSV-G* at native *carO* locus	This study
pMJG42.17978-*carO-VSVG*	Introduction of VSV-G tag at native *carO* locus in ATCC 17978	This study
pMJG42.Δ*ptaZ*	Replacement of *ptaZ* sequence with tR′ terminator	This study
pMJG42.*basJ-VSVG*	Construction of VSV-G tag at native *basJ* locus	This study
pMJG42.Δ*hemZ*	Replacement of *hemZ* sequence with the intrinsic transcription terminator for *rplQ* (*ABUW_RS02125*)	This study
pMJG42.Δ*adeN*	Deletion of *adeN* (*ABUW_RS08430*) open reading frame	This study
pMJG42.Δ*adeIJK*	Deletion of *adeI* (ABUW_RS04135), *adeJ* (*ABUW_RS04130*), and *adeK* (*ABUW_RS04125*) open reading frames	This study
sRNA expression plasmids		
pMJG598	Empty vector, expression controlled by TetR/PtetA	(13)
pAar-FL	pMJG598 with *aar* region inserted, includes Aar promoter	This study
pAar	pMJG598 with *aar* under control of TetR/PtetA	This study
pAar-M1	pMJG598 with M1-allele of *aar* under control of TetR/PtetA	This study
pPtaZ	pMJG598 with *ptaZ* under control of TetR/PtetA	This study
pPtaZ-M1	pMJG598 with M1-allele of *ptaZ* under control of TetR/PtetA	This study
pHemZ	pMJG598 with *hemZ* under control of TetR/PtetA	This study
pHemZ-M1	pMJG598 with M1-allele of *hemZ* under control of TetR/PtetA	This study
CRISPRi plasmids		
pKEV39	*dCas9* Tn7-delivery plasmid	(13)
pMJG440	Control sgRNA plasmid	(13)
pKEV41	*hfq* sgRNA plasmid	(13)
pMJG492	*csrA* sgRNA plasmid	This study
Tn7 Integration plasmids		
pMJG561	Tn7-delivery plasmid for *lacZ* transcriptional and translational fusions	(13)
pMJG609	Tn7-delivery plasmid for *lacZ* transcriptional and translational fusions under *lacIq-Ptac* expression control	(13)
pMJG561.*rsuA::lacZ-TL*	Tn7 plasmid for *rsuA::lacZ* translational fusion	This study
pMJG561.*rsuA::lacZ-TX*	Tn7 plasmid for *rsuA::lacZ* transcriptional fusion	This study
pMJG561.*basJ::lacZ-TL*	Tn7 plasmid for *basJ::lacZ* translational fusion	
pMJG561.*basJ*^{M1C}*::lacZ-TL*	Tn7 plasmid for *basJ::lacZ* translational fusion with compenstatory mutation M1C	This study
pMJG561.*basJ::lacZ-TX*	Tn7 plasmid for *basJ::lacZ* transcriptional fusion	This study
pMJG609.*adeI::lacZ-TL*	Tn7 plasmid for *adeI::lacZ* translational fusion under driven by P*tac* promoter	This study
pMJG609.*adeI*^{M1C}*::lacZ-TL*	Tn7 plasmid for *adeI::lacZ* translational fusion under driven by P*tac* promoter with compensatory mutation M1C	This study

SupplementaryTable S5. – Oligonucleotide Primers used in this study

Primer Name/Function	Sequence (5' -> 3')[a,b]	Source
Chromosomal mutations via pMJG42		
P1_*hfq-VSVG*	cctgcagcccgggggatccactagtGCAGGCGAAAGAAGACTTGCGA	This study
P2_*hfq-VSVG*	aaacggttcatttcaatatccgtgtaagcggccgcACGATTGTTTTCGTCGTCTTGACCATCTTC	This study
P3_*hfq-VSVG*	acggatattgaaatgaaccgtttgggtaagTAATTGATTAGCTTGAAAAAAACCAGTCAGTGATG	This study
P4_*hfq-VSVG*	gtgagagggccgcggtggcggccgCAGCGGCATATCCAAACCAGACAC	This study
P1_*carO-VSVG*	cctgcagcccgggggatccactagtGACAAGAATCTTTGAAAAAACGTGTTACGTTTAATTGAGG	This study
P2_*carO-VSVG*	acggttcatttcaatatctgtgtaagcggccgcCCAGAAGAAGTTCACACCAACTTTACCAAC	This study
P3_*carO-VSVG*	acacagatattgaaatgaaccgtttaggtaagTAATAAAGCATAAAAACGAGCTTCGGCTCGT	This study
P4_*carO-VSVG*	gtgagagggccgcggtggcggccgcGAATTATCCGAGAAACAGTACGCCAAAACTT	This study
P2_*carO-M1C-VSVG*	acacgtaatactttcatcgttttctcct***A***AAGAAAAGGCTCTGTTTTTAATTTATTCAGC	This study
P3_*carO-M1C-VSVG*	GAAAACGATGAAAGTATTACGTGTTTTAGTGAC	This study
P4_*carO-M1C-VSVG*	gtgagagggccgcggtggcggccgcTTATCATCGTTAGCAATTTTACGAGCTTCC	This study
P1_17978-*carO-VSVG*	cctgcagcccgggggatccactagtTGTGACAAGAATCTTTGAAAAAACGTGTTACGT	This study
P2_17978-*carO-VSVG*	acggttcatttcaatatctgtgtaagcggccgcCCAGTAGAAGTTTACACCAACTTTACC	This study
P3_17978-*carO-VSVG*	acagatattgaaatgaaccgtttaggtaagTAATAAATAGTAAAAAAACGAGCTTCGGCT	This study
P1_*basJ-VSVG*	cctgcagcccgggggatccactagtACGTTAAATACACCTGCAGC	This study
P2_*basJ-VSVG*	tcatttcaatatctgtgtaagcggccgcCACAGCAATTTTTGTATTTAGAATATTTAACA	This study
P3_*basJ-VSVG*	gcttacacagatattgaaatgaaccgtttaggTAAGTAATTAGTAAACCTAGGCTGGTTT	This study
P4_*basJ-VSVG*	gtgagagggccgcggtggcggccgcGTTACATGGACATTAGATAGCTTAAAC	This study
P1_Δ*hemZ::rplQ*^{term}	cctgcagcccgggggatccactagtATCTAATCTATTTCGATCATCTTTGAGG	This study
P2_Δ*hemZ::rplQ*^{term}	gaccggcataaagccggtctttttgaatctagaTTAGGCGACTTTTTCTTCTAAACC	This study
P3_Δ*hemZ::rplQ*^{term}	aaaagaccggctttatgccggtctttttattttGATTTCCATTTGCCTTCATTCAAA	This study
P4_Δ*hemZ::rplQ*^{term}	gtgagagggccgcggtggcggccgcTCGATTATGAAATTTTCCGAGAAGTTTA	This study
P1_Δ*adeN*	cctgcagcccgggggatccactagtCCATTTGAAACCCAGACTCGATT	This study
P2_Δ*adeN*	gactttatgtgcggccgcCATAAAATTTAATAACGACGGCTAACACTG	This study
P3_Δ*adeN*	tttatggcggccgcacataaagtcTAATTCTTTTGCATTTTGAAATGC	This study
P4_Δ*adeN*	gtgagagggccgcggtggcggccgcTTAAGCTTTTTTCAAATTTTCGTTGAGTAA	This study
P1_Δ*adeIJK*	cctgcagcccgggggatccactagtGTCTCACCAACTTATTTGTGAGTTTCA	This study
P2_Δ*adeIJK*	aaaattattgcgcggccgcCATCATTGTTCCACCTCGTTTAGATAAAA	This study
P3_Δ*adeIJK*	tgatggcggccgcGCAATAATTTTTAAATCACTTTCTTATAAAAGCTC	This study
P4_Δ*adeIJK*	gtgagagggccgcggtggcggccgcACAGCACAATTTAAGCAATCCTC	This study
sRNA expression plasmids		

Aar-FL_fwd	tcgttgatagagttattttaccttCTTTTACCCTTTTAACGTGAAGTAACACT	This study
Aar_fwd	tcgttgatagagttattttaccttGAGTAGGTTGATATGAACCTCACGACAT	This study
Aar_rev	tcgggattcagccactagtaagcttAAAAAAATACGCAATGATTGGGGTGATCACTG	This study
P2_Aar-M1	TGTCGTGAGGTTCATATCAACCTACTCAAG	This study
P3_Aar-M1	GGTTGATATGAACCTCACGACATTTCTCCT***A***AAGCCGAAACTG	This study
PtaZ_fwd	atcgttgatagagttattttaccttTAATATCATTTAATCTTTTCTACCTCTCGCCA	This study
PtaZ_rev	tcgggattcagccactagtaagcttACCGAAAGTCGAATAACACAAAGG	This study
PtaZ-M1_fwd	atcgttgatagagttattttaccttTAATATCATTTAATCTTTTCT***TG***CTCTCGCCATTT	This study
HemZ_fwd	tcgttgatagagttattttaccttATCGCCCTGAATGGGATGAAGACT	This study
HemZ_rev	tcgggattcagccactagtaagcttGCACTGAATATTCAGCAGGCCTCAC	This study
P2_HemZ-M1	CCAAAGAATCCCCAAAACAAAAAATTAGGCGAC	This study
P3_HemZ-M1	TTTTTTGTTTTGGGGATTCTTTGGCTT***G***CACCCTAAAGAA	This study
lacZ reporter primers		
*rsuA::lacZ*_fwd	catgagctcgaattcccggggatccTTGAGAGCCAAAATGTCAGAGCTTTTAAGGTT	This study
*rsuA::lacZ*_TL_rev	gttgtaaaacgacgggagcaagcttTTGTAAAATTTTTTCCAGCAGCATAAAGCTTTCTC	This study
*rsuA::lacZ*_TX_rev	cgggatcgctagttagttactgcagGCTTTATCATCTTCGCATTTAACTTCA	This study
*basJ::lacZ*_fwd	catgagctcgaattcccggggatccAGTCATGTTGGATCAAAAAAATCATGTTATG	This study
*basJ::lacZ*_TL_rev	gttgtaaaacgacgggagcaagcttAAATTTCTTTTGACCGATATGATCTAAATTTTGC	This study
*basJ::lacZ*_TX_rev	gggatcgctagttagttactgcagTAAGGATATAAAAATACTAAAACACACATTAATGCA	This study
P2_*basJ*^{M1C}*::lacZ*_TL	CATCATCTGTATATC***TG***CTCTCACATTGA	This study
P3_*basJ*^{M1C}*::lacZ*_TL	TCAATGTGAGAG***CA***GATATACAGATGATG	This study
*adeI::lacZ*_TL_fwd	caggaaacagactagtgctctgcagGGCAATGATTATGTTATGCCATAAGCACA	This study
*adeI::lacZ*_TL_rev	gttgtaaaacgacgggagcaagcttTGCGCAAGCAGTAAGGGCTG	This study
P2_*adeI*^{M1C}*::lacZ*_TL	TCGTTTAGATAAAATCTAAAAATATATTTGTTTTCTACTTAACCTACTTTGTG	This study
P3_*ade*^{M1C}*::lacZ*_TL	TATATTTTTAGATTTTATCTAAACGAGGTG***C***AACAATGATGTCG	This study
5' RACE Primers		
PtaZ_RACE	ATTTGAGCGGATATCAAATGGCGAGAG	This study
TSO_RT	GCTAATCATTGCAAGCAGTGGTATCAACGCAGAGTACATRGRGRG	This study
TSO_NESTED	CATTGCAAGCAGTGGTATCAAC	This study
qRT-PCR Primers		
*recA*_fwd	CGCAAACTTGGTGTAGATATTG	This study
*recA*_rev	TTAAATCAATTGCGCCTGAAC	This study
*adeI*_fwd	TGCAGAAGTAGGTGTTATTGTT	This study
*adeI*_rev	GGACGAACTTCAGAAATTTGATATG	This study
*adeJ*_fwd	GGTAGATTGAATGGCACAATTT	This study
*adeJ*_rev	TAAGAATACCCGCCAACATAAT	This study
*adeK*_fwd	ATCAGATTACACAGAACAACCA	This study
*adeK*_rev	TTACACCCAAACCTACTTGATAA	This study
Probes for Northern Blotting		
5S rRNA	/5IRD700/GGTTTCACTTCTGAGTTCGGGAAGG	This study
Aar	TACGCAATGATTGGGGTGATCACTGCGTAG	This study

sRNA99	CCACATCCTTCTTCTCGTTTTACTCACTTCCG	This study
PtaZ	GCGGATATCAAATGGCGAGAGGTAGA	This study
HemZ	GGAGATTCTTTAGGGTGGAAGCCAAAGAAT	This study
OmpZ	CCTCACATAAAAAACTATGTGAGGTGCCTGAA	This study
sRNA32	CATCTTGGGGAAAATGTAGGCTATAAAATG	This study
sgRNA expression plasmid		
*csrA*_sgRNA_fwd	gctcagtcctaggtataatactagtCAATCATTAATGTTTCCCCGACAgttttag agctagaaatagcaag	This study
sgRNA_reverse_p440	CAGATCCTCTTCTGAGATGAGTTTTTGTTCGG	(13)

Notes: a) lower case letters indicate homology regions added to the 5′ end of primers to facilitate recombination-based cloning

b) Nucleotides listed in bold/Italic/underlined font indicate the mutations being introduced

Supplementary Table S6. Synthetic gBlocks used in this study

gBlock	Sequence (5′ -> 3′)

∆aar::tR′

CCTGCAGCCCGGGGGATCCACTAGTAACGTAACCCAACCATCAAATCTGAAATTCAAATGCGTGGTTTTGAAC
GTGATATTCCTGCCGGTTTCCTTTGCTACCCTGTAGCACAGGCAGCTGATATTACGGCTTTTAAAGCGACTGT
AGTACCAGTAGGTGAAGATCAAATTCCAATGATTGAGCAAACTAACGAAATCGTGCGTCGTGTAAACCGACAA
ATTGGTCAAGACCTTTTACCTGAATGTAAAGCTTTGCTTTCTAATATGGCTCGTTTACCTGGTTTTGATGGTAAA
GCGAAAATGTCTAAGTCATTAGGCAATACCATTGTTTTAAATGCTTCTGACAAAGACATCAAAAAAGCTGTAAA
TGCGATGTATACCGATCCAAATCATCTTCGCATCGAAGATCCGGGCCAAGTTGAAGGAAACATTGTATTTACT
TATTTAGATGCATTTGATCCAAATAAAGAAGAAGTTGAAGAGCTAAAAGCACACTACCGCCGTGGTGGTCTTG
GTGATGGTACAGTTAAAAAACGTCTTGAAGGTGTACTAAAAGAATTAATTACACCTATACGTGAACGTCGTGAA
GAACTTGCTAAAGATCCTGACTATATTATGGATGTTTTACGCCAAGGTACTGATAAATGTCGAATCATTACCCA
GCAGACTTTGGACGAAGTTAAAGATGGTTTAGGCTTATTTAAGTTCTAATTTTTATATTAGAGATATAAATCAAA
AAAGCCCTACGGGGCTTTTTTTATAATTATTTTTAGATTCAACAATTTAATTTTTTAGAATTTCTATATATTTATTA
TGATTCACACGTCCCGAAACAGTTCGCAGGTAATAGTTAGAGCCTGCATAACGGTTTCGGGATTTTTTGTCTA
TAATAATGTTGGCAATTTAGGTTGAAATTCAACCGAGTTATAATAATAATTCTGTTGAAAATACTCAAACTACTT
TGTCTGATCAAGTGGTGAATAGCGCGTTACACTATCACTCACAAGCGCCTTTGTTTGATAACTTAATGAATGAA
TTGGGCTTACGGTTGTGAGGTCAATGGTTTGACTCAAGGGTTGAAACCAAAAGCTGAGTAAGGATGTACACTC
TGTTTAGTACTCCTATTTCAATGTAGACCGAAATGCTAAATATTTGATCTACAATTTTCATCTTCCGTTTCGTCA
ATTCCTATGGTGAAAATGAGTAATTAGATACCAAGTTCAGTATAAGGTTAGCAAAATATCAAACAGGGCATCTC
TCCCCTTGAATTAAATCACGCGTTGAAAAAGTATAGCGAAACAGATGACCAACATTTAATAAGAGAGGCGCTG
ACTACAAGCGACCTCTCGATGGATGAATAAACGTGATGGACTAAGCTTTATGGACTAGAAGGTGGTAGTAAAG
AAATTTGTTTAAGAGGCTCGCGTTTTGCCCCAAGTTCGCGAAGCTTTTCCATAAAACTAGATTCCTTTATTGGT
AGACCACCAATGATTTTCCCTACTTCAGCGTTGATGATAGCAGCAACAATCAATCCACCAATTGCTCCCCCTG
CAGCGCCAGCTCCTGTAGCTTGACCGTAGCTCATATTTCCTGAGGAAAAGTTCTTACCAATTATGGCACCAGT
AACACCTCCTACTGTGCCAGCTGTGGCAAAGGATACTAGACTAACTTTATGGGATGCGGCCGCCACCGCGGC
CCTCTCAC

∆ptaZ::tR′

CCTGCAGCCCGGGGGATCCACTAGTGTTACCGGCCGGGTTAGAAATTATTGATCCAGATCAGATCCGTGACC
AATATATTGCACCAATGGTTGAACTCCGCAAAGGCAAACTCAACGACCTACAAGCTAAAGAGCAATTACAAGA
TACTGTAGTTCTCGGCACCATGATGCTAGCTCTAGATCATGTGGATGGATTAGTTTCAGGGGCAGTTCATACA
ACCGCCAATACAGTGCGTCCAGCTTTCCAATTGATTAAAACCGCTCCAGCATACTCACTGGTTTCATCCATTTT
CTTTATGCTTTTACCGGATGAAGTCTATGTCTACGGTGACTGTGCAATTAACCCAGACCCTACAGCCGAACAG
CTTGCCGAAATTGCGATTCAGTCTGCTGACTCTGCAAAAGCGTTTGGAATTGATCCACGTATTGCCATGATCA
GCTACTCAACTGGTACATCTGGAACAGGTGCTGATGTTGAGAAAGTACAGCAAGCGACTCAAATTGCACAGC
AACGTCGCCCTGATTTACTGATTGACGGTCCACTACAATACGATGCGGCTTCAGTTGAAAGTGTAGGACGCCA
AAAGGCGCCAGACTCACGCGTTGCTGGCCGTGCCAATGTATTTATTTTCCCAGACTTAAATACGGGTAACACG
ACTTACAAAGCAGTACAACGTGCAGCAAATGTAGTTAGCGTTGGCCCAATGCTGCAAGGCCTAAATAAGCCA
GTCAATGACTTGTCTCGTGGTGCTTTAGTAGATGACATCGTCTTTACTATCGCATTAACAGCCATTCAGGCCG
AGCAGCAAGCTGCTGCCAAATAATTCCCGAAACAGTTCGCAGGTAATAGTTAGAGCCTGCATAACGGTTTCG
GGATTTTTTATTTAAAAAAATAAGCCTGATTCTAGGTTGTTGTTAATTTTAAATAACTTAGCCTTTGTGTTATTCG
ACTTTCGGTCAAAAGATCAACCAATCCCCCACAAGATTGTCTTTTTGTCATATAATTTAGCCCGCTAGCTAACA
GCCCGCTCAAGAGATTTTTTGGTATGACAACGATTATCAAACAAGATGACTTGATTACATCAATCAAGGACGC
CCTACAGTTTATTTCGTACTATCACCCGCAAGACTTTATCCAAGCGATGAGCCGTGCTTATGATCGCGAAGAA
AACAAAGCTGCAAAGGATGCAATTGCACAGATTTTAATTAACTCTCGCATGTGTGCGGAAGGTCATCGTCCAA
TTTGCCAAGATACTGGTATTGTTAACGTTTTCCTTGAAGTGGGCTTAGATGTTAAATTTGATTTAACAATGAGCT
TAGACGATGCAGTAAATGAAGGTGTTCGCCAAGGTTACCTTGAAAACAGCAACGTTCTTCGTGCATCTGTTCT
TGCTGACCCTGCATTTGGTCGTAAAAATACAAAAGACAATACCCCTGCCGTTATTCACTACAAACTCGTACCA
GGTAACAAAGTAGATATTACTGTTGCTGCTAAAGGTGGCGGTTCAGAAAACAAATCTAAACTTGCGATGCTTA
ACCCATCTGACTCGATTGTTGACTGGGTTCTTAAAACCGTTCCAACTATGGGTGCAGGCTGGTGTCCTCCTGG
TATGCTCGGTATCGGTATTGGCGGTACTGCTGAAAAAGCCATGATGCTTGCGAAAGAAGCACTCATGGAAGA
AGCGGCCGCCACCGCGGCCCTCTCAC

Supplementary Methods

Plasmid Construction
All plasmids created in this work (Supplemental Table S2) via isothermal assembly (14). Briefly, parental plasmids were digested with the appropriate restriction enzymes (New England Biolabs) and purified by column clean up following gel electrophoresis. PCR products were amplified using the primers indicated in Supplemental Table S3 using KODX polymerase (Millipore Sigma), resolved by agarose electrophoresis and purified using Qiagen Gel Extraction columns according to the manufacturer's recommendations. For ITA, reactions contained 1X ITA reaction mix, linearized vectors, and purified PCR products and were assembled on ice, incubated at 50˚C for 15-30 minutes, and transformed by heat-shock into chemically competent DH5α F′IQ cells. The resulting plasmids were confirmed by dideoxy-sequencing (Iowa State University DNA Facility) or whole plasmid sequencing (Plasmidsaurus) prior to use in *A. baumannii*.

The allele exchange plasmid, pMJG42, confers resistance to tetracycline and sensitivity to sucrose, used to create chromosomal mutations in *A. baumannii* (8). All pMJG42 derivatives created in this work were constructed using ITA with plasmid pMJG42 that had been digested with NotI and SpeI. Plasmid pMJG42.*hfq-VSVG* was used to create a chromosomally encoded *hfq* allele specifying the Vesicular Stomatitis Virus-G protein epitope (VSV-G) at the C-terminus of the *hfq* coding sequence, just prior to the stop codon. The plasmid was created by combining linearized pMJG42 plasmid with PCR products created by amplification of AB5075-UW genomic DNA with primers P1_*hfq-VSVG* & P2_*hfq-VSVG* and primers P3_*hfq*-VSVG & P4_*hfq*-VSVG. Plasmid pMJG42.*carO-VSVG* was used to create a chromosomally encoded *carO* allele specifying the VSV-G epitope at the C-terminus of the *carO* coding sequence, just prior to the stop codon. The plasmid was created by combining linearized pMJG42 plasmid with PCR products created by amplification of AB5075-UW genomic DNA with primers P1_*carO-VSVG* & P2_*carO-VSVG* and primers P3_*carO-VSVG* & P4_*carO-VSVG*. Plasmid pMJG42.Δ*aar* was used to create a deletion mutation of *aar* where the Aar sequence was replaced with the tR' terminator sequence. The plasmid was created by combining linearized pMJG42 plasmid with the Δ*aar::tR'* gBlock product (synthesized by IDT). Plasmid pMJG42.*carO-M1C-VSVG* was used to introduce the M1C-allele of *carO* allele, also specifying a VSV-G epitope at the C-terminus of the *carO* coding sequence. The plasmid was created by combining linearized pMJG42 plasmid with PCR products created by amplification of AB5075-UW genomic DNA with primers P1_*carO-VSVG* & P2_*carO-M1C-VSVG* and primers P3_*carO-M1C-VSVG* & P4_*carO-M1C-VSVG*. Plasmid pMJG42.17978-*carO-VSVG* was used to create a chromosomally encoded *carO* allele specifying the VSV-G epitope at the C-terminus of the *carO* coding sequence, just prior to the stop codon in the *A. baumannii* ATCC 17978 background. The plasmid was created by combining linearized pMJG42 plasmid with PCR products created by amplification of ATCC-17978 genomic DNA with primers P1_17978-*carO-VSVG* & P2_17978-*carO-VSVG* and primers P3_17978-*carO-VSVG* & P4_*carO-M1C-VSVG*. Plasmid pMJG42.Δ*ptaZ* was used to create a deletion mutation of *ptaZ* where the PtaZ sequence was replaced with the tR' terminator sequence. The plasmid was created by combining linearized pMJG42 plasmid with the Δ*ptaZ::tR'* gBlock product (synthesized by IDT). Plasmid pMJG42.*basJ-VSVG* was used to create a chromosomally encoded *basJ* allele specifying the VSV-G epitope at the C-terminus of the *basJ* coding sequence, just prior to the stop codon. The plasmid was created by combining linearized pMJG42 plasmid with PCR products created by amplification of AB5075-UW genomic DNA with primers P1_*basJ-VSVG* & P2_*basJ-VSVG* and primers P3_*basJ-VSVG* & P4_*basJ-VSVG*. Plasmid pMJG42.Δ*hemZ* was used to create a *hemZ*-deletion mutation where the HemZ sequence was replaced with the intrinsic transcription terminator for *rplQ* (ABUW_RS02125 // ABUW_0432). The plasmid was created by combining linearized pMJG42 plasmid with PCR products created by amplification of AB5075-UW genomic DNA with primers P1_Δ*hemZ::rplQ*^{term} & P2_Δ*hemZ::rplQ*^{term} and primers P3_Δ*hemZ::rplQ*^{term} & P1_Δ*hemZ::rplQ*^{term}. Plasmid pMJG42.Δ*adeN* was used to create an in-frame deletion of the open reading frame of *adeN* (*ABUW_RS08430 // ABUW_1731*). The deletion construct was generated to replace the bulk of the *adeN* coding sequence; the resulting open reading frame included the *adeN* start codon and three alanine residues followed by the final three amino acids of the *adeN* coding sequence and the stop codon. The plasmid was created by combining linearized pMJG42 plasmid with PCR products created by amplification of AB5075-UW genomic DNA with primers P1_Δ*adeN* & P2_*adeN* and primers P3_Δ*adeN* & P4 Δ*adeN*. Plasmid pMJG42.Δ*adeIJK* was used to create an in-frame deletion of *adeI* (ABUW_RS04135), *adeJ* (*ABUW_RS04130*), and *adeK* (*ABUW_RS04125*). The resulting open reading frame included the first two codons of *adeI*, three alanine residues, and the final codon of *adeK* coding sequence and the *adeK* stop codon. The plasmid was created by combining linearized pMJG42 plasmid with PCR products created by amplification of AB5075-UW genomic DNA with primers P1_Δ*adeIJK* & P2_*adeIJK* and primers P3_Δ*adeIJK* & P4 Δ*adeIJK*.

For sRNA expression, we used the incompatibility group Q plasmid, pMJG598, which encodes apramycin resistance and has an anhydrotetracycline-reponsive promoter (TetR-P_{tetA}) positioned immediately upstream of a multiple cloning site (13). All sRNA expression constructs were clone into the parent plasmid at the DraIII and HindIII restriction sites on the parent plasmid. Plasmid pAar-FL, which encodes for Aar with 80 nucleotides of upstream DNA to capture Aar promoter. The plasmid was created by combining linearized pMJG598 with PCR a product created by amplification of AB5075-UW genomic DNA with primers Aar-FL_fwd and Aar-rev. Plasmid pAar, encoding wild-type Aar sRNA, was created by combining linearized pMJG598 with a PCR product created by amplification of AB5075-UW genomic DNA with primers Aar-fwd and Aar-rev. Plasmid pAar-M1, which encodes an allele of Aar with a single nucleotide change (T36A) that disrupts the interaction of Aar with the *carO* mRNA, was created by combining linearized pMJG598 with PCR products created by amplification of AB5075-UW genomic DNA with primers Aar-fwd & P2_Aar-M1 adnd P3_Aar-M2 & Aar-rev. Plasmid pPtaZ encodes for the sRNA PtaZ; the PtaZ expression construct is 103 nucleotdies long and begins immediately following the *pta* stop codon and continues past the PtaZ transcription terminator for an additional ≈ 40 nt, 5′-RACE experiments indicate that PtaZ begins 20 nucleotides downstream of the *pta* stop codon. The plasmid was created by combining linearized pMJG598 with a PCR product created by amplification of AB5075-UW genomic DNA with primers PtaZ_fwd & PtaZ_rev. Plasmid PtaZ-M1, which encodes an allele of PtaZ with two nucleotide changes (A2T and C3G) that disrupts the interaction of PtaZ with the *basJ* mRNA, was created by combining linearized pMJG598 with a PCR product created by amplification of AB5075-UW genomic DNA with primers PtaZ-M1_fwd & PtaZ_rev. Plasmid pHemZ encodes for the sRNA HemZ and includes upstream sequence corresponding to the last 98 nucleotides of the *hemF* open reading frame (including the *hemF* stop codon) and an additional 78 nucleotides downstream of the predicted HemZ transcription terminator sequence. The pHemZ plasmid was created by combining linearized pMJG598 with a PCR product created by amplification of AB5075-UW genomic DNA with primers HemZ_fwd & HemZ rev. Plasmid pHemZ-M1 encodes for a mutant allele of HemZ where HemZ position 28 was changed from a G to a C nucleotide. The plasmid was created by combining linearized pMJG598 with PCR products created by amplification of AB5075-UW genomic DNA with primers HemZ_fwd & P2_HemZ-M1 and P3_HemZ-M1 & HemZ_rev.

For *lacZ* fusion plasmids, we integrated the reporter constructs at the Tn7 attachment site as previously described (13). Plasmid pMJG561 is a Tn7-delivery plasmid and contains a promoterless *lacZ* gene and restriction cloning sites allowing for the construction of either transcriptional or translational *lacZ* fusions (13). Plasmid pMJG609 is a derivative of pMJG561 where a $lacI^{q}$-P_{tet} regulation system drives expression of the *lacZ* fusions (13). All derivatives of pMJG561 and pMJG609 described herein were created by ITA. For *rsuA* and *basJ* translational *lacZ* fusions in pMJG561, the plasmid was linearized BamHI and HindIII. For the *rsuA* and *basJ* transcriptional fusions in pMJG561, the plasmid was linearized with BamHI and PstI. For the *adeI* translational *lacZ* fusion, plasmid pMJG609 was digested with PstI and HindIII. Plasmid pMJG561.*rsuA::lacZ-TL* contains an in-frame translational fusion between the *adeI* open reading frame with the 8th codon of *lacZ* and was created by amplifying a 554-bp region including the *rsuA* 5′-UTR up to the 24th bp of the *rsuA* open reading frame with primers *rsuA::lacZ*_fwd & *rsuA::lacZ*_TL_rev which was combined with linearized pMJG61 via ITA. Plasmid pMJG561.*rsuA::lacZ-TX* contains the predicted promoter region upstream of *rsuA* and was created by combining linearized plasmid pMJG561, via ITA, with a PCR product comprising a 466-bp region of the *rsuA* 5′-UTR that was PCR amplified with primers *rsuA::lacZ_fwd* & *rsuA::lacZ*_TX_rev, which was combined with linearized pMJG561 via ITA. The 466-nucleotide *rsuA* promoter construct ends at a position 64 nucleotides upstream of the *rsuA* start codon. Plasmid pMJG561.*basJ::lacZ-TL* contain an in-frame translational fusion between the *adeI* open reading frame with the 8th codon of *lacZ* and was created by PCR amplifying a 607-bp region including the *basJ* 5′-UTR up to the 60th bp of the *basJ* open reading frame with primers *basJ::lacZ*_fwd and *basJ::lacZ_TL_rev*, which was combined with linearized pMJG561 via ITA. Plasmid pMJG561.*basJ::lacZ-TX* contains the predicted promoter region upstream of *basJ* and was created by PCR amplifying a 485-bp region of the *basJ* 5′-UTR with primers *basJ::lacZ_fwd* & *basJ::lacZ_TX_rev*, which was combined with linearized pMJG561 via ITA. The 485-nucleotide *basJ* promoter region construct ends at a position 62 nucleotides upstream of the *basJ* start codon. Plasmid pMJG561.*basJM1C::lacZ-TL* contains the same *basJ::lacZ* translational fusion as pMJG561.*basJ::lac-TL*, except for a two-nucleotide mutation at the complementory position to the PtaZ-M1 mutation which corresponds to positions -11 (G changed to C) and -10 (T changed to A) relative to the translation start site of *basJ.* The plasmid was created by PCR amplifying two fragments with primers *basJ::lacZ_fwd* & *P2_basJ*^{M1C}*::laZ_TL* and *P3_basJ*^{M1C}*::lacZ_TL* & *basJ::lacZ_TL_rev* and combining with linearized pMJG561 via ITA. Plasmid

pMJG609.*adeI::lacZ-TL* contains an in-frame translational fusion between the *adeI* open reading frame with the 8th codon of *lacZ* and was created by amplifying a 295-bp region including the *adeI* 5′-UTR up to the 45th bp of the *adeI* open reading frame with primers *adeI::lacZ_TL_fwd* & *adeI::lacZ_TL_rev,* which was combined with linearized pMJG609 via ITA. Plasmid pMJG609.*adeI^{M1C}::lacZ-TL* contains the same *adeI::lacZ* translational fusion as pMJG609.*adeI::lacZ-TL*, except for a single nucleotide mutation at the complementory position to the HemZ-M1 mutation which corresponds to position -5 (G changed to C) relative to the tralsation start site of *adeI*. The plasmid was created by PCR amplifying two fragments with primers *adeI::lacZ_TL_fwd* & *P2_adeI^{M1C}::lacZ-TL* and *P3_adeI^{M1C}::lacZ-TL* & *adeI::lacZ_TL_rev*, which were combined with linearized pMJG609 via ITA.

For CRISPRi experiments, sgRNA constructs were expressed from plasmid pMJG440 and its derivatives. The control sgRNA plasmid, pMJG440 (targeting the mCherry coding sequence) and *hfq* plasmid (pKEV41) have been described previously (13). For targeting *csrA*, we inserted PCR product amplified with primers *csrA_sgRNA_fwd* and *sgRNA_reverse_p440* into plasmid pMJG440 that had been digested with SpeI & ApaI using ITA as described above. The resulting sgRNA targets the non-template strand of the *csrA* open reading frame.

Supplemental References

1. Melamed S, Faigenbaum-Romm R, Peer A, Reiss N, Shechter O, Bar A, Altuvia Y, Argaman L, Margalit H. 2018. Mapping the small RNA interactome in bacteria using RIL-seq. Nat Protoc 13:1-33.
2. Gebhardt MJ, Farland EA, Basu P, Macareno K, Melamed S, Dove SL. 2023. Hfq-licensed RNA-RNA interactome in *Pseudomonas aeruginosa* reveals a keystone sRNA. Proc Natl Acad Sci U S A 120:e2218407120.
3. Hofacker IL. 2003. Vienna RNA secondary structure server. Nucleic Acids Res 31:3429-31.
4. Johnson PZ, Simon AE. 2023. RNAcanvas: interactive drawing and exploration of nucleic acid structures. Nucleic Acids Research 51:W501-W508.
5. Krzywinski MI, Schein JE, Birol I, Connors J, Gascoyne R, Horsman D, Jones SJ, Marra MA. 2009. Circos: An information aesthetic for comparative genomics. Genome Research doi:10.1101/gr.092759.109.
6. Mann M, Wright PR, Backofen R. 2017. IntaRNA 2.0: enhanced and customizable prediction of RNA–RNA interactions. Nucleic Acids Research 45:W435-W439.
7. Hamrock FJ, Ryan D, Shaibah A, Ershova AS, Mogre A, Sulimani MM, Ben Taarit S, Reichardt S, Hokamp K, Westermann AJ, Kröger C. 2024. Global analysis of the RNA-RNA interactome in *Acinetobacter baumannii* AB5075 uncovers a small regulatory RNA repressing the virulence-related outer membrane protein CarO. Nucleic Acids Res 52:11283-11300.
8. Gebhardt MJ, Gallagher LA, Jacobson RK, Usacheva EA, Peterson LR, Zurawski DV, Shuman HA. 2015. Joint Transcriptional Control of Virulence and Resistance to Antibiotic and Environmental Stress in *Acinetobacter baumannii*. mBio 6.
9. Zurawski DV, Thompson MG, McQueary CN, Matalka MN, Sahl JW, Craft DW, Rasko DA. 2012. Genome sequences of four divergent multidrug-resistant *Acinetobacter baumannii* strains isolated from patients with sepsis or osteomyelitis. J Bacteriol 194:1619-20.
10. Poirel L, Menuteau O, Agoli N, Cattoen C, Nordmann P. 2003. Outbreak of Extended-Spectrum β-Lactamase VEB-1-Producing Isolates of *Acinetobacter baumannii* in a French Hospital. Journal of Clinical Microbiology 41:3542-3547.
11. Longo B, Pantosti A, Luzzi I, Placanica P, Gallo S, Tarasi A, Di Sora F, Monaco M, Dionisi AM, Volpe I, Montella F, Cassone A, Rezza G. 2006. An outbreak of *Acinetobacter baumannii* in an intensive care unit: epidemiological and molecular findings. J Hosp Infect 64:303-5.
12. La Forgia C, Franke J, Hacek DM, Thomson RB, Jr., Robicsek A, Peterson LR. 2010. Management of a multidrug-resistant *Acinetobacter baumannii* outbreak in an intensive care unit using novel environmental disinfection: a 38-month report. Am J Infect Control 38:259-63.
13. Intorcia V, Sava RL, Schroeder GP, Gebhardt MJ. 2024. A series of vectors for inducible gene expression in multidrug-resistant *Acinetobacter baumannii*. Appl Environ Microbiol 90:e0047424.
14. Gibson DG, Young L, Chuang RY, Venter JC, Hutchison CA, 3rd, Smith HO. 2009. Enzymatic assembly of DNA molecules up to several hundred kilobases. Nat Methods 6:343-5.
